# Application of the Transcriptional Disease Signature (TDSs) to Screen Melanoma-Effective Compounds in a Small Fish Model

**DOI:** 10.1038/s41598-018-36656-x

**Published:** 2019-01-24

**Authors:** Yuan Lu, William Boswell, Mikki Boswell, Barbara Klotz, Susanne Kneitz, Janine Regneri, Markita Savage, Cristina Mendoza, John Postlethwait, Wesley C. Warren, Manfred Schartl, Ronald B. Walter

**Affiliations:** 1 Xiphophorus Genetic Stock Center, Department of Chemistry and Biochemistry, 419 Centennial Hall, Texas State University, San Marcos, TX USA; 2 Developmental Biochemistry, Biozentrum, University of Würzburg, Würzburg, Germany; 3 Comprehensive Cancer Center Mainfranken, University Clinic Würzburg, D-97074 Würzburg, Germany; 4 Hagler Institute for Advanced Studies and Department of Biology, Texas A&M University, College Station, USA; 5 Institute of Neuroscience, University of Oregon, Eugene, Oregon USA; 6 University of Missouri, Columbia, Missouri USA

## Abstract

Cell culture and protein target-based compound screening strategies, though broadly utilized in selecting candidate compounds, often fail to eliminate candidate compounds with non-target effects and/or safety concerns until late in the drug developmental process. Phenotype screening using intact research animals is attractive because it can help identify small molecule candidate compounds that have a high probability of proceeding to clinical use. Most FDA approved, first-in-class small molecules were identified from phenotypic screening. However, phenotypic screening using rodent models is labor intensive, low-throughput, and very expensive. As a novel alternative for small molecule screening, we have been developing gene expression disease profiles, termed the Transcriptional Disease Signature (TDS), as readout of small molecule screens for therapeutic molecules. In this concept, compounds that can reverse, or otherwise affect known disease-associated gene expression patterns in whole animals may be rapidly identified for more detailed downstream direct testing of their efficacy and mode of action. To establish proof of concept for this screening strategy, we employed a transgenic strain of a small aquarium fish, medaka (*Oryzias latipes*), that overexpresses the malignant melanoma driver gene *xmrk*, a mutant *egfr* gene, that is driven by a pigment cell-specific *mitf* promoter. In this model, melanoma develops with 100% penetrance. Using the transgenic medaka malignant melanoma model, we established a screening system that employs the NanoString nCounter platform to quantify gene expression within custom sets of TDS gene targets that we had previously shown to exhibit differential transcription among *xmrk-*transgenic and wild-type medaka. Compound-modulated gene expression was identified using an internet-accessible custom-built data processing pipeline. The effect of a given drug on the entire TDS profile was estimated by comparing compound-modulated genes in the TDS using an activation Z-score and Kolmogorov-Smirnov statistics. TDS gene probes were designed that target common signaling pathways that include proliferation, development, toxicity, immune function, metabolism and detoxification. These pathways may be utilized to evaluate candidate compounds for potential favorable, or unfavorable, effects on melanoma-associated gene expression. Here we present the logistics of using medaka to screen compounds, as well as, the development of a user-friendly NanoString data analysis pipeline to support feasibility of this novel TDS drug-screening strategy.

## Introduction

The cost of creating and bringing a single new drug to clinical use is estimated to be ≈$5 billion dollars. This high cost is due to the fact that 95% of experimental medicines ultimately fail in human trials due to poor efficacy and/or safety concerns^1^. Late failure occurs because most current drug discovery pipelines focus on protein targets thought to play a key role in disease etiology without consideration of collateral cellular, organ-level, or whole-body changes^2^. This target-based strategy (i.e., identifying substances that alter a specific biochemical activity *in vitro*) has the advantage that it is hypothesis driven, amenable to computational modeling, and is suitable for high-throughput screening of compound libraries, often coupled with cell culture. The number of innovative, FDA-approved medicines, however, has not increased commensurate with monetary investment and most potential compounds fail before approval^3,4^ and exhibit a correlation with a decreased reliance on phenotypic screens^5,6^.

In contrast to target based drug discovery, phenotypic screens have the advantage of not requiring prior understanding of the molecular mechanism of disease, and can therefore cast a broad net, far beyond our current knowledge of disease etiology. A recent analysis of agents approved by the FDA between 1999 and 2008 showed that among first-in-class small molecule drugs, 62% came from phenotypic screens and only 38% from target-based tactics^7^. The success of phenotypic screens compels a renewed emphasis on their development and use for screening potential therapeutic molecules. Although cell-based, target-based discovery assays have rapid throughput they do not adequately predict the compound’s absorption, distribution, metabolism, excretion and toxicity (ADMET). Alternatively, phenotypic screens that employ intact organisms have the great advantage of immediately uncovering ADMET problems, the step at which most potential drugs frequently fail^7^.

In spite of the advantages of using phenotypic screens with intact animal, this strategy is expensive given the cost of housing mammals at numbers required for such assays. However, the use of small aquarium fish can address this barrier for identifying potentially therapeutic drugs. Numerous studies have proven fish to be good vertebrate disease models and small fish models have been utilized to successfully identify drugs that are currently in clinical trials or already in the marketplace^8–35^. Unfortunately, current technologies generally limit fish screens to embryonic stages and restrict incubation times to a few days, while most human diseases may occur in fully developed individuals and often intensify with age. Moreover, chronic diseases, such as diabetes, heart disease, schizophrenia, and cancer require long-term studies and are thus not amenable to current embryo screening tactics. Additionally, although sophisticated automated screening systems exist for certain disease models (e.g., bone mineralization disorders, neurological disorders, stress, amyotrophic lateral sclerosis, cardiovascular disease, mental illness)^8,30,35–40^, traditional phenotypic screens using fish embryos or juveniles require manual scoring of phenotypic changes. This time-consuming process limits throughput of this strategy. Therefore, with the goal of improving the drug development pipeline, we have proposed to improve phenotypic drug screening by a novel approach; (1) to utilize disease-related gene expression patterns or signatures – termed Transcriptional Disease Signatures, or TDSs – as a phenotype to be scored, and (2) to identify compounds that are able to shift gene the expression signature toward a different state as a first pass screen.

This proposed strategy has several advantages over other methods. First, aquarium fish models (i.e., medaka, zebrafish) are small (i.e., ~10 mm at 4 weeks of age), allowing them to fit in a single well of multi-well plates, which allows convenient housing of large numbers of animals required for mid- to high-throughput screening, and fit with automated sample handling system. Second, the cost to maintain aquaria fish species is much less than to maintain rodents. Third, compounds to be tested can be applied directly to the water that bathes the animal, allowing one to assess bioavailability with properly labeled compound once a hit is found. Transcriptional changes are likely to represent some of the earliest phenotypes altered by drugs, occurring well before–and in fact, usually causing–the onset of later visible phenotypes. Therefore, identifying compounds capable of modulating the TDS phenotype may lead to promising drug candidates for further testing and clinical trials. Fourth, currently, it is very difficult to screen animals at different life stages, however, If TDS screening is found valuable, it could be adapted to any age of the animal, with an acceptable decline in throughput simply due to size of later stage animals.

To establish the TDS-based screening strategy, resolve logistics, and construct data analysis tools, we utilized a transgenic melanoma medaka model to institute a drug-screening pipeline. The medaka melanoma model expresses the melanoma driver transgene termed the *Xiphophorus* Melanoma Receptor Kinase (*xmrk*), that is regulated by pigment cell-specific *mitf* (melanogenesis associated transcription factor) promoter^41,42^. The *xmrk* oncogene is a mutant copy of fish Epidermal Growth Factor Receptor (EGFR) identified in the platyfish *Xiphophorus maculatus*^43^; *xmrk* overexpression in transgenic medaka leads to tumor development with 100% penetrance. The oncogenic transgene drives the development of pigment cell lesions and exhibits genetic background-specific tumor types when incorporated into different lines of medaka. For example, the *xmrk* transgene results in cutaneous exophytic xanthoerythrophoroma in *Cab* line, invasive extracutaneous melanotic melanoma, and exophytic xanthoerythrophoroma in *Carbio* line, uveal melanoma in albino *i-3* line, and extracutatneous invasive melanotic melanoma in *HB32C* line^41,42^. Expression of *xmrk* drives several proliferation pathways that are also involved in human cancers^44–48^. Genes regulated by *xmrk* in medaka and *Xiphophorus* melanoma are implicated in networks that also characterize human melanoma, so the *xmrk-*associated gene expression profile represents the human melanoma transcriptional phenotype^41,49–52^. Additionally, fish and human melanocytes both appear in the epidermis, while mouse melanocytes occupy hair follicles^49^. These attributes make *xmrk-*transgenic medaka a good model to screen potential anti-melanoma compounds and to establish the logistics of using the TDS concept as a screening tool.

**Figure 1:**
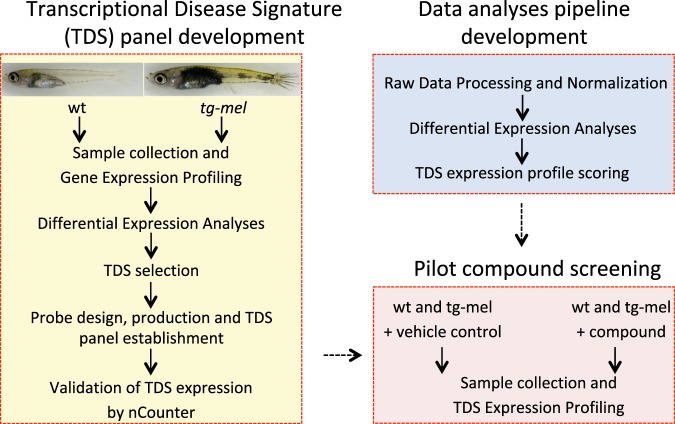
TDS development pipeline and drug screening flow chart. The proposed compound-screening pipeline includes three phases: the identification of Transcriptional Disease Signature (TDS) genes, the development of data analyses tools, and the testing of the established screening pipeline using compounds that are commonly used for melanoma therapy.

In this study, we establish a TDS pipeline using whole transcriptome profiling of wild-type (wt) and *xmrk-*transgenic (*tg*-*mel*) medaka to identify target genes, construct NanoString probe sets targeting an identified TDS genes, and test two compounds to establish the feasibility of this drug screening approach (Fig. 1).

## Materials and Methods

### Fish Utilized

Three-week-old wild-type (wt) and *xmrk-transgenic* (*tg*-*mel*), also designated tg(*mitf:xmrk*), from the Cab strain [for a detailed description of genotypes and phenotypes see^42^] were raised in the *Xiphophorus* Genetic Stock Center. For TDS testing, both *tg*-*mel* and wt medaka were euthanized for whole-fish RNA isolation in accordance with an approved Institutional Animal Care and Use Committee (IACUC) protocol (IACUC20173294956). Texas State University has an Animal Welfare Assurance on file with the Office of Laboratory Animal Welfare (OLAW), National Institute of Health. The assurance number is #A4147.01. For NanoString nCounter confirmation of TDS expression and compound screening, *tg*-mel and wt medaka were individually maintained in 96-well-plates (one fish/well) for 24 hrs prior to drug treatment. Fish were fed daily with freshly hatched brine shrimp (~10 brine shrimp/fish). The water, along with test compounds, was changed daily.

### Small molecule treatment

Trametinib (GSK1120212) was purchased from Selleckchem (Catalog Number S2673) and Cisplatin was purchased from Calbiochem (Catalog Number 232120). Trametinib was dissolved in DMSO and stored in −20 °C. Cisplatin powder was stored at 4 °C. 0.5 mg/mL (1.67 mM) Cisplatin stock solution was freshly made by dissolving in 0.9%(w/v) NaCl solution for each day of treatment.

In Drug Trial 1, five *tg*-mel and four wt medaka were used as vehicle controls. Two *tg*-mel medaka were treated with 25 nM Trametinib; Three *tg*-mel medaka were treated with 50 µM Cisplatin. Treatments lasted three days with compound and water refreshed daily. In Drug Trial 2, three *tg-*mel were used as vehicle controls, three *tg-*mel were treated with 25 nM Trametinib, and three *tg-*mel were treated with 50 µM for three days.

### Design of Transcriptional Disease Signatures

To establish a TDS target gene set for compound screening, we first performed transcriptome profiling of both wt and *tg*-mel medaka fish. Ten intact wt and 10 *tg*-mel medaka fish at age between 3–4 weeks, with body length of around 10 mm were anesthetized by placing them on ice, sacrificed, and then immediately placed in 1.5 mL microcentrifuge tubes containing 300 µL TRI Reagent (Sigma Inc., St. Louis, MO, USA) followed by flash freezing in an ethanol dry ice bath. Whole fish were homogenized with a tissue homogenizer while still frozen in TRI Reagent. After the initial homogenization, 300 µL of fresh TRI Reagent and 120 µL of chloroform were added to the 1.5 mL microcentrifuge tube and shaken vigorously for 15 sec. Phase separation was performed by centrifugation (12,000 × g for 5 min at 4 °C). The aqueous phase was then added to a new 1.5 mL microcentrifuge tube and an additional chloroform extraction was performed (300 µL TRI Reagent, 60 µL chloroform). Following extraction, the nucleic acids were precipitated with 500 µL of 70% EtOH and transferred to a Qiagen RNeasy mini spin column. DNase treatment was performed on-column for 15 min at 25 °C, and RNA samples were subsequently eluted with 100 µL RNase-free water. RNA concentrations were quantified with a Qubit 2.0 fluorometer (Life Technologies, Grand Island, NY, USA), and RNA quality was assessed based on RNA integrity (RIN) score with an Agilent 2100 Bioanalyzer (Agilent Technologies, Santa Clara, CA, USA).

All samples sequenced were required to have RNA Integrity (RIN) score ≥ 8.0. Individual sequencing libraries were constructed using the Illumina TruSeq mRNA Library Prep Kit with polyA selection, and libraries were sequenced (100 bp, paired-end [PE] reads) on the Illumina HiSeq 2000 platform. RNA sequencing and raw reads filtering was performed as previously described^53–57^. Sequencing adaptors were trimmed from raw reads, and subsequent short sequencing reads were filtered using a custom Perl script^58^ that removed low scoring sections of each read while preserving the longest remaining fragment (for statistics of RNA-Seq, Table S1).

To take advantage of different gene expression profiling pipelines, in order to increase the ability to identify comprehensive Differentially Expressed Genes (DEGs) between the *tg-mel* and wt medaka, processed sequencing reads were mapped to the medaka reference genome (Ensembl release 85, ftp://ftp.ensembl.org/pub/release-85/fasta/oryzias_latipes/dna/) using Tophat2^59^ or STAR^60^. The percent of reads mapped and level of coverage were calculated by dividing total length of sequencing reads by the total length of all exons (Table S1). Gene expression was quantified using FeatureCounts following Tophat2^61^ or RSEM after alignment using the STAR aligner^62^. DEGs between *tg*-*mel* and wt medaka were identified using R/Bioconductor edgeR package^63,64^ or DESeq 2^65^. The Area Under Curve (AUC) of the Receiver Operating Characteristic (ROC) curve was calculated to assess true and false positive rates for each gene tested by the R package pROC. A set of statistical thresholds was applied to define TDS: log_2_Fold Change (log_2_FC) ≥ 0.7 or ≤−0.7, and False Discovery Rate (FDR) ≤ 0.05; AUC ≥ 0.7. A total of 222 genes met these criteria and were designated as the preliminary TDS that are subject to validation using further Nanostring analyses. In addition to these genes, 29 genes that were expressed at a variety of levels and did not show differential expression between *tg*-mel and wt medaka were manually selected as housekeeper genes. Also, 108 genes were included as TDS to represent several functional categories related to the compounds to be tested. These genes were selected to represent several different pathways: cell proliferation, circadian regulation, DNA repair, and common toxicology related genes (For final probe set details, see Table S3). A capture probe and a reporter probe were designed to quantify one gene of interest. Specifically, a capture probe contained two portions of specific sequence. One was complementary to a unique sequence on the targeted transcript, and the other was complementary to a reporter probe. Once a capture probe was bound to the targeted transcript, and further bound by a reporter probe, the probe-transcript complex was fixed on a silicone surface. The quantification of each transcript was made possible by counting the light signal generated by a series of fluorophores that specifically coded each transcript and attached to the reporter probe. Design and production of the reporter and capture probes was performed by the NanoString bioinformatics group (NanoString, Seattle, WA). Transcript sequences corresponding to each gene target were downloaded from Ensembl (www.ensembl.org) as templates for probe design. Each probe was 100 nt long, with a melting temperature between 73 and 91 °C and did not form secondary structures that could lead to assay inhibition. Probes were also tested *in silico* to avoid cross hybridization to other loci.

To validate the TDS gene expression data, and finalize selection of TDS genes, twenty 3-week old wt and twenty 3-week old *tg*-mel medaka were placed in individual wells of a 96-well plate (one fish per well), maintained for a week with daily feeding of brine shrimp and water changes, and finally sacrificed at 4-weeks old. As a repeat, another fifteen 3- to 4-week-old wt and fifteen age matched *tg-mel* were also sacrificed for TDS gene expression assessment. Total RNA from whole fish was isolated and TDS gene expression was assayed using the NanoString nCounter system. Log_2_FC, and AUC values of ROC curve of each TDS, were calculated in each test. Of the RNA-Seq identified 222 TDS genes, 97 showed consistent direction of Log_2_FC between *tg-mel* and wt in two separate tests, and AUC > 0.7 in at least one test. These 97 genes were weighted differently to reflect their expression pattern within the *tg-mel* and wt medaka populations, and were forwarded within the TDS for further testing. Twenty-three genes that have ROC curve AUC values > 0.8 in each of the two tests were given a weight of two. For the rest of the 74 genes, their weights were determined by the AUC values of the ROC curves. Twenty-one genes were weighted between 0.9 and 1, 33 genes were weighted between 0.8 and 0.9, and 20 genes were weighted between 0.7 and 0.8.

### Correlation and Principle Component Analysis

Spearman ranking correlation was performed using R programming correlation function. Principle Component Analysis (PCA) was performed using the R package prcomp, and normalized RNA-Seq gene expression read counts, or nCoutner TDS probe counts were used in Spearman ranking correlation and PCA. A heatmap of correlation coefficients and dendrograms were plotted using the gplots R package.

### Construction of the data analysis pipeline

To facilitate the processing of large quantities of data, we constructed a data analysis pipeline to normalize samples from different batches, identify differentially expressed genes, and evaluate compound effects on changing the TDS expression. The pipeline included three internet-accessible user interfaces (UIs) hosted at the *Xiphophorus* Genetic Stock Center (XGSC). The UIs can be launched directly through the XGSC website (www.xiphophorus.txstate.edu/TDSproject.html), or using command line through R interface. The latter method will download the UI package through Github and configure local workstation as a server to host the UI. For detailed description of the UI usage, please see: www.xiphophorus.txstate.edu/TDSproject.html.

The normalization user interface, called “TDSNormalization” first assessed NanoString nCounter hybridization efficiency by calculating the mean of built-in positive controls (i.e., pre-loaded short oligonucleotides) that generated fragment counts independent of sample RNA, and by normalizing gene expression levels targeted by custom probes by multiplying a normalization factor that is inversely related to the hybridization efficiency. This normalization step removed cartridge-specific hybridization differences among assays performed on different days, and therefore allowed comparison of data generated over the experimental time line. Next, the normalization user interface calculated the geometric mean of the expression in housekeeper genes and further normalized gene expression counts by multiplying a sample-specific scaling factor that removes effects caused by differences in total RNA input. Subsequently, background noise was calculated by the mean (μ) and standard deviation (σ) of built-in negative control probes. Sample-specific background noise was determined to be μ + 2 × σ and was subtracted from expression counts generated from custom probes.

The Differentially Expressed Gene (DEG) Identification user interface, called “DEGAnalysis”, took normalized gene expression counts as input, and calculated effective size using mean and standard deviation of user-defined samples. The DEG Identification user interface subsequently calculated Log_2_Fold Change (Log_2_FC), and performed Welch two-sample t-test (two-sided) and calculated p-value. These statistical parameters are included in a UI-generated gene expression report.

Next, a Drug Score user interface, named “TDSScore”, calculates two parameters that evaluate the effect of tested compounds on TDS expression phenotypes. Activation Z-score is used to determine whether a compound worked as an activator of the disease-like state (i.e., changes TDS expression pattern to a disease status) or a repressor of the disease-like state (i.e., shifts TDS expression towards a wild-type status) by counting the number of genes that show consistent and contradicting expression changes compared to a list of reference genes (i.e., TDS). Briefly, we took a statistical approach by defining a Z-score that determined whether a compound has significantly more “activated” TDS than “repressed” TDS (Z > 0) or vice versa (Z < 0). Here, significance means that we rejected the hypothesis that the overall effect of the compound on TDS expression is random with equal probability. The distribution underlying this null hypothesis is defined by a random variable:

$${{x}}_{{\rm{i}}}\in \{-1,1\}$$

where +1 corresponded to a consistent state and −1 to a contradicting state, and both values were chosen with probability 1/2. The index i ran from 1 to N with N being the number of TDS. Let:

$$x={\sum }_{{\rm{i}}}{x}_{{\rm{i}}}={N}_{+}-{N}_{-}$$

where *N*_+/*−*_ was the number of “activated”/ “inhibited” TDS and *N*_+_ + *N*_*−*_ = *N*. The variance of *x*_i_ is σ^2^ = 1, so the variance of x was given by:

$${\sigma }_{x}^{2}=N{\sigma }^{2}=N$$

and the Z-score statistic (with mean equal to zero and variance equal to 1) is defined by:

$$Z=x/{\sigma }_{x}={\sum }_{{\rm{i}}}{x}_{{\rm{i}}}/\sqrt{N}=({N}_{+}-{N}_{-})/\sqrt{N}$$

A Z-score greater than 2 or smaller than −2 is considered significant.

Because Z-score statistics did not take into account the degree of each probe’s differential expression, the Kolmogorov-Smirnov statistic (Ks_drug score) was also utilized in the TDSScore UI, to estimate and rank the effectiveness of compounds in “activating” or “repressing” TDS expression with consideration of which TDS genes a compound modulates^66–69^. For each compound treatment, the Kolmogorov-Smirnov statistic is computed for both up-regulated genes and down-regulated genes in the TDS, giving *ks*^*i*^_*up*_ and *ks*^*i*^_*down*_. Let *n* be the number of the TDS and *t* be the number of compound-related differentially expressed genes. The UI first ordered all *n* TDS by the extent of their expression change after drug treatment, then constructed a vector *V* of the position (1… *n*) of each differentially expressed gene in the ordered list of all TDS, and next sorted these components in ascending order such that *V(j)* was the position of differentially expressed gene *j*, where *j* = 1, 2, …, *t*. The UI finally computed the following two values:

$$a=\,\max [\frac{j}{t}-\frac{V(j)}{n}]$$

$$b=\,\max [\frac{V(j)}{n}-\frac{(j-1)}{t}]$$

If *a* > *b*, set *ks* = *a*. If *b* > *a*, set *ks* = −*b*. The up scores and down scores were *ks*_*up*_ and *ks*_*down*_, respectively. These values were reported in the results table as “up” and “down”, respectively. The Ks_drug score *S* is set to zero where *ks*_*up*_ and *ks*_*down*_ have the same sign. Otherwise, set *S* to be *ks*_*up*_−*ks*_*down*_.

### Automated RNA isolation and NanoString assay

Medaka fish were sacrificed by placing the whole fish directly in 750 μL of QIAzol (Qiagen) in 2.0 mL collection tubes designed for automated (i.e., 96 well) tissue homogenization and RNA isolation (Qiagen, TissueLyser II). Whole fish were homogenized using the TissueLyser II (Qiagen) facilitated by stainless beads (Qiagen) for 10 min at 25 hz. RNA isolation was subsequently performed using a QIAcube HT (Qiagen) automated bio-sample isolation system. The isolation system is equipped with a robotic arm with 8 pipettes. Each pipette is able to pick and eject pipette tips, self-clean, and transfer liquids between wells/columns, or between master reservoirs and wells/columns in standard 96-well plate formats (Fig. S1). Each sample was independently maintained throughout the isolation process. Briefly, 150 µL of chloroform was added to each isolation tube and the samples were vigorously shaken for 15 sec and then phases partitioned by centrifugation (12,000 × g for 15 min at 4 °C). The aqueous phase containing nucleic acids was transferred to a new sample tube by a rack of automated pipettors. After extraction, nucleic acids were precipitated with 500 µL 70% EtOH. RNA was then purified using a Qiagen RNeasy mini RNA kit (96-well plate) following the manufacturer’s protocol. RNA was quantified with a Qubit 2.0 fluorometer (Life Technologies, Grand Island, NY, USA). Concentrations of RNA samples were adjusted to 100 ng/μL with RNase-free water (Qiagen).

NanoString hybridization of RNA samples with the TDS panel was initiated by mixing 500 ng of RNA (100 ng/µL) with the custom designed NanoString capture and reporter probe sets. Samples were incubated for 12 hrs at 65 °C and then processed by the NanoString Prep Station (NanoString Technologies, Seattle, WA, USA). The NanoString cartridge containing the hybridized samples was immediately evaluated with the NanoString nCounter based on unique color-coded signals. Probe counts were quantified through direct counting with the nCounter Digital Analyzer. Probe counts generated from individual samples were downloaded from the nCounter system and were combined into a count table using a custom R script. Raw probe reads were normalized to built-in positive controls and processed (i.e., normalization to housekeeper genes, removal of background noise) using the normalization UI for further analyses.

At the end of each drug trial, RNA was isolated from whole fish, and gene expression was assayed using NanoString nCounter platform as described above. Raw gene expression counts were normalized using the Normalization UI, and DEGs were analyzed using DEG Identification UI. NanoString sensitivity has been detected accurately and reproducibly down to a 20% change^70^, therefore DEGs were identified by comparing *tg*-mel to wt medaka, compound treated wt to control wt medaka, or compound-treated *tg*-mel to control *tg*-mel medaka (log_2_FC ≥ 0.6 or ≤ −0.6, *p*-value ≤ 0.05). Drug Score UI was used to determine the compound effect on shifting TDS gene expression.

### Functional analyses of differentially expressed genes

Human orthologs of medaka genes were identified using the Ensembl database with the R Biomart package. Signal pathway(s) and functional categories were retrieved from Ingenuity Pathway Analyses (IPA) software (Qiagen, Redwood City, CA). Multiple representations of pathway and functional categories due to several human-medaka homolog genes pairs were combined.

## Results

### Establishing the Transcriptional Disease Signature

**Figure 2:**
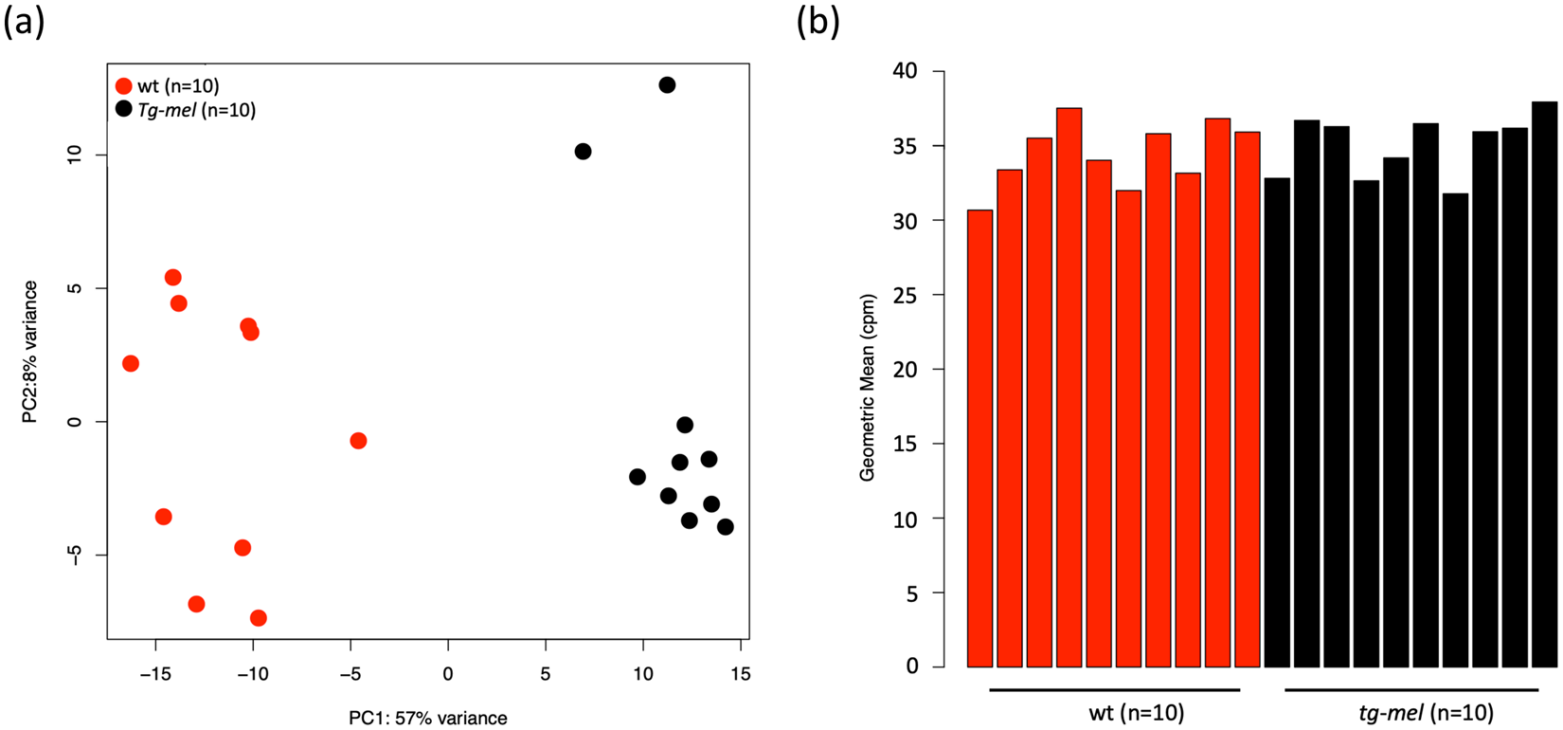
TDS genes selected from gene expression profiling of *tg*-mel medaka. (**a**) Principle Components Analysis shows that TDS genes account for 57% of variance between *tg*-mel and wt. *Tg*-mel and wt samples can be clearly separated based on the expression profile of TDS genes. (**b**) Geometric means of housekeeping genes in each sample are similar in expression level between wt and *tg-mel* medaka.

Gene expression profiling in ten wt and ten *tg*-mel medaka and differential gene expression analyses identified 222 DEGs between wt and *tg*-mel medaka. These genes composed our TDS panel to be tested on the NanoString nCounter and represented putative transcriptional phenotype differences between wt and *tg*-mel medaka (Table S2). As proof of concept, further validation of this preliminary TDS gene panel was carried out. First, our PCA using the preliminary TDS genes clearly segregated the wt and *tg*-mel medaka fish cohort (Fig. 2a). To control for the amount of input RNA in the subsequent Nanostring nCounter assay, we show 29 genes (housekeeping classification) did not show significant differential expression over all wt and *tg*-mel medaka (Fig. 2b). Additionally, we included 108 genes in the preliminary TDS panel that are involved in liver toxicity, DNA repair, detoxification, cell cycle regulation, and pre-proliferation pathways (Table S3), to simultaneously measure their responses to the compounds tested.

**Figure 3:**
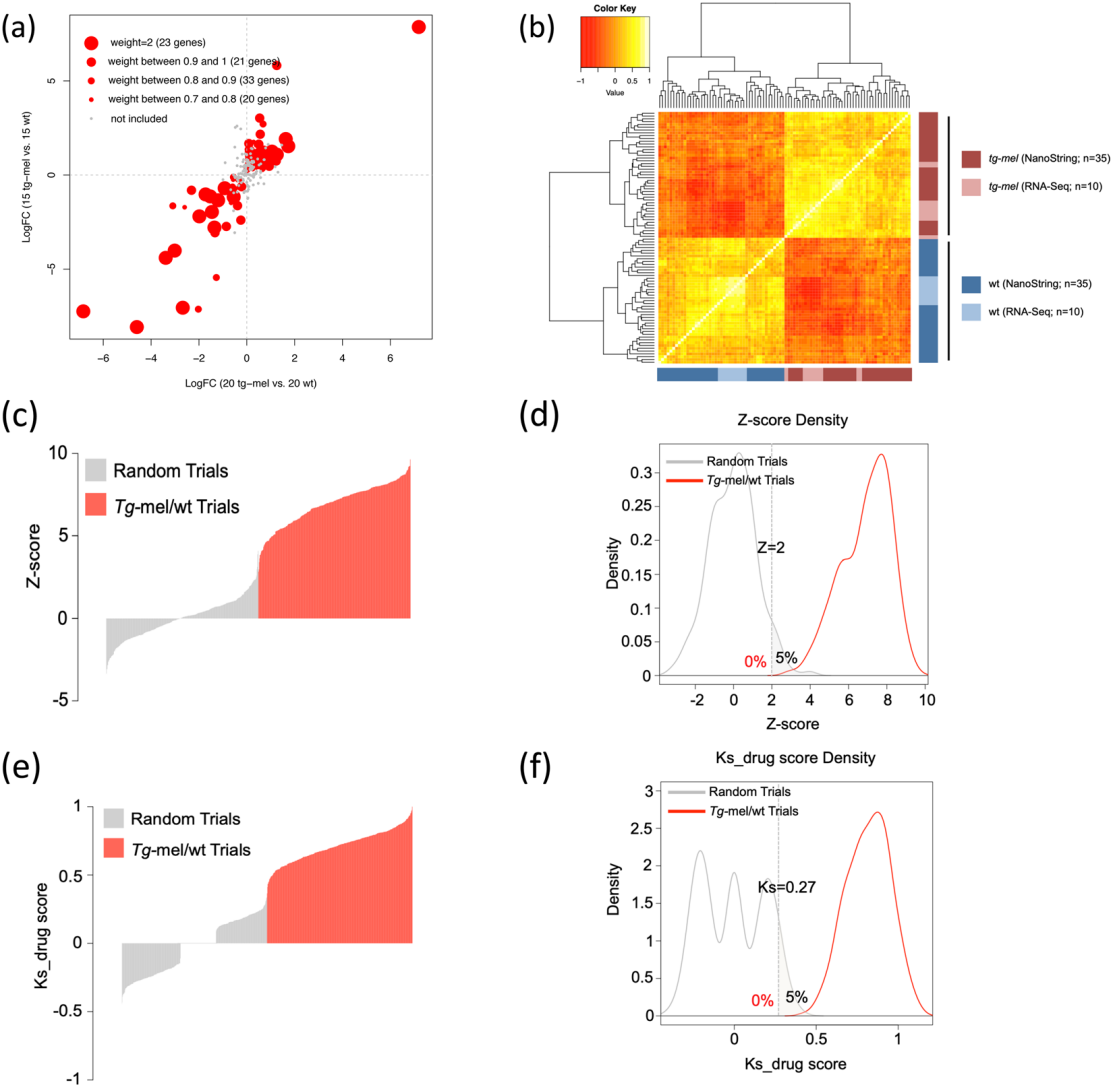
Gene expression profiling of TDS between RNA-Seq and nCounter. (**a**) Of 222 genes identified as TDS by RNA-Seq, 97 showed a consistent direction of differential expression between *tg-mel* and wt in two separate tests. These 97 genes were weighted differently to reflect their expression patterns within the *tg-mel* and wt medaka populations and were retained as TDS genes for further test. Twenty three of these 97 genes were given a weight of two because their AUC values of ROC curve in each of the two tests were above 0.8. Weights of the remaining 74 genes were determined by the AUC values of the ROC curves. (**b**) Spearman ranking correlation analysis was performed on RNA-Seq data for ten *tg*-mel fish and ten wt individuals and two independent NanoString nCounter assessed medaka samples (a total of 35 wt and 35 *tg-mel* medaka). Genotypes (i.e., *tg*-mel or wt) clustered together independent of methodology. (**c**–**f**) The TDS gene expression pattern itself (i.e., Reference TDS) serves as a standard to calculate the TDS expression pattern; a reversed pattern of Reference TDS simulates a compound that can make each TDS gene return to a non-diseased expression level (i.e., Model compound). The TDS expression profile of each of 20 *tg*-mel individuals was compared to that of each of 20 wt medaka, resulting in 400 possible comparisons. Using the Log_2_FC values generated in these 400 comparisons, 400 incidences were simulated by randomly choosing Log_2_FC values to estimate the false positive and false negative rate of Z-score and Ks_drug score statistics. (**c**) Weighted TDS activation Z-scores were calculated for the Reference TDS, model compounds, 400 simulated datasets and 400 TDS expression patterns. (**d**) A Z-score of two resulted in 0% false negatives and 5% false positives in identifying TDS activation status. (**e**) The Ks_drug score was calculated for the Reference TDS, model compounds, 400 simulated datasets and 400 TDS expression patterns. (**f**) A Ks_drug score of 0.27 resulted in 0% false negatives and 5% false positives in identifying TDS activation pattern.

After having constructed the custom NanoString panel and synthesized target gene-specific probes, we performed targeted transcript counting on two sets of independent medaka samples, which included both *tg-mel* and wt medaka, aiming to validate the TDS genes using an independent assay, and to limit the TDS to a set of reliable genes that are capable of distinguishing transcriptional phenotypes using Nanostring platform. A total of 97 of the 222 preliminary TDS gene targets showed the same direction of differential expression between the two Nanostring tests, with ROC curve AUC value in at least one test larger than 0.7, and were determined to be the final TDS for compound screening (Fig. 3a). The final Nanostring panel consists of 234 probes, including 97 probes that target the TDS, 29 housekeeping genes, and 108 genes that belong to various selected pathways considered to be important to screening (Table S3). As a quality control of TDS probes, we normalized and processed the probe counts using TDS Normalization UI. Subsequently the gene expression profiles established by NanoString were compared to the expression profile assessed by RNA-Seq that were performed on independent animals. Ranking correlation showed that wt and *tg*-*mel* samples clustered separately independent of technology used to assess gene expression levels (Fig. 3b).

### Evaluation of the Transcriptional Disease Signature

Having established that TDS genes were capable of distinguishing *tg-mel* and wt medaka, we next evaluated the effectiveness of using Z-score and Ks_drug score to quantify the transcriptional phenotype in order to use this to evaluate drug treatment effects. Here we proposed to 1) test whether Z-score and Ks_drug score algorithms assign positive scores (i.e., consistent with TDS reference expression pattern) to diseased animals; 2) estimate the false discovery rate of using the scoring algorithms.

To fully represent TDS relative expression in known diseased to healthy individuals, we calculated Log_2_FC values of each TDS gene between each *tg-mel* to each wt medaka, and acquired 400 lists of Log_2_FC values of TDS genes. These lists of values represent all possible TDS relative expression patterns from a population of 20 wt and 20 *tg-mel* medaka. Each list of the values was subsequently used to calculate a Z-score and a Ks_drug score. These lists of values resulted in a mean Z-score of 6.9 (Fig. 3c), and a mean Ks_drug score of 0.7 (Fig. 3e). Using the Log_2_FC value matrix, we have also created 400 lists of values by randomly selecting Log_2_FC values to represent scenarios where no diseased individuals are present in dataset, or where treatment with a drug did not lead to directional changes of TDS expression. These second data matrices were used to assess possible Z-score and Ks_drug scores from noise. The 400 lists containing randomized Log_2_FC values resulted in a mean Z-score of 0, so did the Ks_drug score (Fig. 3c,e).

Next we used the distribution of Z-scores and Ks_drugs score, calculated as detailed above, to estimate false positive rate. Randomized data only had a 5% chance to reach a Z-score of 2, or Ks_drug score of 0.27 (Fig. 3d,f). On the other hand, 100% of Z-scores and Ks_drug scores calculated from the data matrix that contained *tg-mel/*wt TDS Log_2_FC led to values larger than 2 or 0.27 respectively (Fig. 3d,f). Therefore, we used |Z-score| ≥ 2 and |Ks_drug score| ≥ 0.27 as thresholds to determine if a drug treatment led to a transcriptional phenotypic change.

### Small Molecule Treatment and Effect

After establishment of the TDS panel, next we examined whether compound altered gene expression pattern can be captured by the screening system. Because Trametinib and Cisplatin are used to treat melanoma, we selected them to test the effectiveness of the TDS-based pipeline for screening compounds. Trametinib is an inhibitor of MEK1 and MEK2^71,72^, which are involved in the Ras/Raf/MEK/ERK(MAPK) signal transduction cascade. Inhibiting the signaling cascade with MEK1/2 inhibitors leads to clinical benefits for treatment of cancers with dysregulation of this pathway. Expression of *xmrk* activates the MAPK pathway and drives the dedifferentiation of melanocytes^44^. Thus, Trametinib was used in this study as a control compound targeting a direct downstream effector of the *xmrk* transgene. Cisplatin is a broadly used chemotherapy agent in several types of cancer, including melanoma^73,74^. Its mechanism of action involves inter-strand DNA crosslinking, induction of DNA repair, interference with DNA replication, and initiation of apoptosis. Cisplatin was included in the pilot study to test whether the TDS-screening strategy can identify this compound as a potent anti-cancer agent.

**Figure 4:**
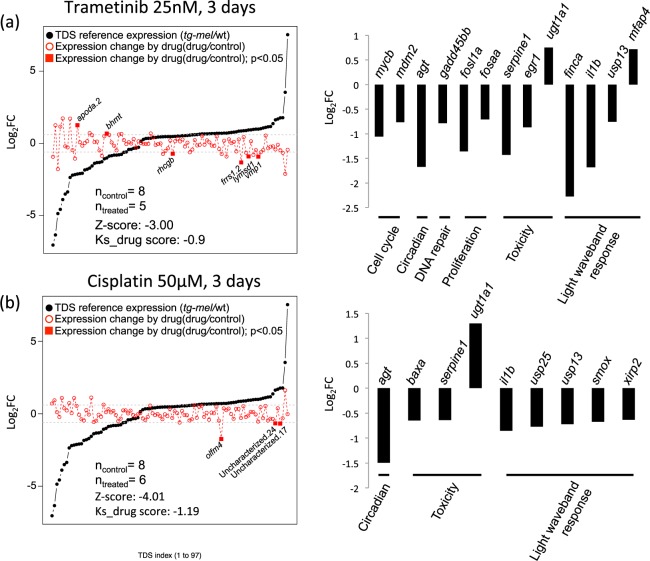
Evaluations of Trametinib and Cisplatin in TDS expression. Trametinib and Cisplatin were used to test the TDS screening system. In each plot, differential expression of TDS genes between *tg-mel* and wt medaka were plotted in ascending order of TDS Log_2_FC values (black dots). Gene expression changes after drug treatments were calculated by comparing the gene expression of drug-treated *tg-mel* to vehicle-treated *tg-mel*. Log_2_FCs by drug were plotted as open red dots in the order of reference TDS gene expression. Solid red dots represent statistically significant (*p-value* < 0.05; |Log_2_FC| ≥ 0.6) differentially expressed genes by drug treatment between drug-treated *tg-*mel fish and control individuals *tg-*mel. Differentially expressed genes that belong to reference pathways and functions are plotted in the bar graph. Genes are grouped in functions and plotted in ascending order of Log_2_FC by drug treatment. (**a**) Of the 97 TDS genes representing the transcriptional phenotypic difference between *tg-mel* and wt medaka, 25 nM Trametinib altered the expression of six TDS genes, and 13 genes belonged to reference functional categories. Drug scoring calculation resulted in a TDS activation Z-score of −3.00, and a Ks_drug score of −0.9. Eight control samples consisted of five control samples from trial 1, and 3 from trial 2; five drug-treated samples consisted of two samples from trial 1 and three from trial 2. (**b**) 50 µM Cisplatin treatment led to differential expression of three TDS genes, and nine genes belonged to reference functional categories in *tg*-mel medaka. Cisplatin treatments resulted in a TDS activation Z-score of −4.01, and Ks_drug score of −1.19. Eight control samples consisted of five control samples from trial 1, and three samples from trial 2; six drug-treated samples consisted of three samples from trial 1 and three samples from trial 2.

A total of eight 3-week-old *tg*-mel medaka (five in trial 1; three in trial 2) were used as vehicle controls for Trametinib and Cisplatin treatment. Five (two in trial 1; three in trial 2) age-matching *tg*-*mel* medaka were treated with 25 nM Trametinib (Fig. 4a). Six 3-week-old *tg*-mel medaka (3 in trial 1; 3 in trial 2) were treated with 50 µM Cisplatin respectively for three days (Fig. 4b). Drug scoring calculations resulted in TDS activation Z-scores of −3.00, and Ks_drug score of −0.90 for 25 nM Trametinib treatments (Fig. 4a). Cisplatin at 50 µM concentration resulted in TDS activation Z-score of −4.01, and Ks_drug score of −1.19 (Fig. 4b). Twenty-five nM Trametinib treatments altered the expression of six genes (*apoda*.*2*, *bhmt*, *rhcgb*, *frrs1*.*2*, *lymsd1*, *vmp1*; Fig. 4a). Treatments with 50 µM Cisplatin led to differential expression of 3 TDS genes in the *tg-mel* medaka (Fig. 4b). Some of the TDS genes that exhibited transcriptional response to these drug treatments serve as a proliferation markers in the mouse melanoma model (*apoda*.*2*), as well as, early stage colorectal cancer, metastasis of breast cancer (*olfm4*), prognostic indicators in human hepatocellular carcinoma (*bhmt*), regulators of cancer cell migration and apoptosis (*rgcgb*), or directly involved in pancreatic cancer (*vmp1*)^75–82^. These observations suggest that TDS expression patterns may be applied to screens in other animal modes and human disease.

## Discussion

Among molecular disease markers, such as gene expression, protein expression, patterns of DNA methylation, and metabolite profiles, gene expression is the most broadly used marker to measure gene activity due availability of cost-effectiveness of methods that assess levels of RNA. Additionally, transcript levels, compared to other parameters, is a direct reflection of genome status. Using gene expression to study disease mechanisms and applying disease-specific gene expression markers to identify potential treatment compounds has received long-standing interest. For example, Hughes *et al*. demonstrated that functions of small molecules can be explained by a collection of gene expression signatures^83^. Most strategies to capitalize on the relationship between gene expression and disease have utilized *in vitro* systems because experimentation with intact animals, especially mammalian experimental models, is expensive^84^. Coupling transcriptional phenotype changes with assessment in intact animals to select promising compounds for potential therapies has not been widely used on a mid- to high-throughput scale. This screening strategy, however, once established, can become a useful additional component in the drug development pipeline, as a step between target, cell-culture based screening strategies, and mammalian model-based preclinical tests. The TDS screening system detailed herein serves as a first step toward combining the advantages of both *in vitro* target-based screening and *in vivo* phenotypical screening.

This study aims to establish the logistics of TDS-based screening system. We used a transgenic medaka melanoma (*tg*-mel) model as an example, to identify gene expression changes, or TDS, that represented a diseased status, and known anti-melanoma agents as test compounds to examine whether gene expression change by drug treatment can be captured using the proposed system. Schartl *et al*. established the *tg*-mel medaka by expressing the fish oncogene *xmrk* under *mitf* control for expression in medaka pigment cells^42^. Previous expression profiling on wt and *tg*-mel medaka showed differential expression of 338 genes that have human homologues^85^. Forty-one of these 338 genes matched human genes previously shown to contain putative deleterious variants in human melanoma samples. A detailed canonical pathway evaluation showed significant enrichment for 50 diverse pathways, including melanocyte development and pigmentation, leukocyte signaling and antigen pattern recognition. The similarity between *tg*-mel and human melanoma renders *tg*-mel medaka a superb transcriptional representation of human melanoma^49,52,85^.

We established the medaka melanoma TDS gene set by identifying the most representative genes differentially expressed between wt and melanoma-bearing *tg*-mel medaka using RNA-Seq based transcriptome profiling. One common observation, especially when using animal-based samples, is the variation in signal readout among different individuals. The sources of gene expression variance are likely due to environment factors, genetic background (e.g., eQTLs), or stochastic gene expression^86^. In our experience, although we observed reproducible differential expression of TDS genes in different datasets, we also observed that not all *tg-*mel medaka showed the same over-expression/under-expression pattern for all TDS genes. Instead of limiting the transcriptional phenotype to only a handful of genes that show absolute expression consistency among sample populations, we weighted each gene’s contribution to the overall phenotype by the likelihood that a gene target would show a differential expression pattern in diseased individuals. This way, when a compound appears to regulate a TDS gene, a potentially therapeutic compound’s effect is also weighted by how prevalent the given gene is disease-representative in the diseased population. Incorporating the weighting procedure in the screening system has the advantage of allowing the identification of compounds that may be effective in modulating disease subtypes and also allows the TDS to represent a large number of distinct cellular pathways.

To evaluate the reliability of the TDS panel, transcript counts generated by NanoString nCounter were compared to differential expression in RNA-Seq experiments. The clustering of the NanoString nCounter-generated TDS profile and the RNA-Seq-generated TDS profile from wt and *tg*-mel medaka, suggests that TDS genes can identify transcriptome states that differentiate medaka, with or without the *xmrk* transgene (Fig. 3b). This observation supports the use of gene expression signatures and direct transcript quantification from intact animals. Z-score and Kolmogorov-Smirnov (Ks) statistics were shown capable of identifying disease-associated transcriptional phenotypes given levels of gene expression variation displayed by medaka test populations, with a false discovery rate of 5% (Fig. 3).

The analysis of NanoString data requires a large amount of command line input and installation of a number of software packages. To streamline data analysis and facilitate the application of a pipeline for bench-top scientists, we developed a software suite that performs routine normalization, differential expression analysis, and calculation of activation Z-score and Ks_drug score. The pipeline is accessible through XGSC website, or locally by R interface through running R scripts stored in Github (For details: see www.xiphophorus.txstate.edu/TDSproject.html). Normalization, DEG identification, and Drug-score calculation were all performed using the UI, thus supporting its feasibility within this new phenotypic screening pipeline.

To further test the TDS screening system, we performed a pilot study aiming to determine: 1) whether the TDS screening pipeline can identify gene expression changes caused by treatments with putative therapeutic compounds; and 2) whether anti-cancer compounds that are used in clinical applications show promising therapeutic effect in an intact animal. Two compounds, Trametinib and Cisplatin, are commonly used to treat melanoma patients. Given the *xmrk* oncogene is reported to suppress differentiation of mouse melanocytes by maintaining MAPK activation^44^ we expected Trametinib, as a MEK1/2 inhibitor, to repress some TDS genes associated with MAPK signaling, because MEK1/2 are closely related and regulate the Ras/Raf/MEK/ERK signaling cascade. We also anticipate some of our TDS genes belong to common carcinogenesis pathways and that toxic responses may show altered treatment responses. Although these genes do not necessarily represent transcriptional differences between normal and diseased individuals, they can be used to test potential compound activity on specific signaling pathways, and to roughly evaluate toxicity of a tested compound. Including these gene targets in the TDS enhances the output derived from the TDS screening strategy. Expression changes in TDS genes after drug treatment are directly used to test whether compounds can alter disease-associated transcriptional phenotypes. The presence of DEGs after drug treatment, and repressed TDS expression pattern showed that both expectations were met. Additionally, the transcriptional change of genes targeting reference pathways provides information on how a given compound affects various cellular processes. This information can help determine the potential usage of a compound in other disease models, evaluate side effects, and characterize molecular mechanisms of action. For example, Trametinib, in addition to the TDS genes, also affected the proliferation-related gene targets *fosl1a* and *fosaa*, and toxicity related genes *serpine1*, *egr1* and *ugt1a1*. It is noted that both Trametinib and Cisplatin down-regulate vascular toxicity related *serpine1*, and up-regulate detoxification-related *ugt1a1* (Fig. 4). This observation may suggest that Trametinib treatment exhibits similar toxicity as hallmarked by these two genes^87,88^. Although using transcriptional markers may not be conclusive for determination of toxicity of the tested compounds, identification of toxicity related transcriptional responses serves as an early warning message, indicating the potential for toxicity that needs to be tested in more detail.

This study focuses on using coding gene transcriptional phenotypes as bait to evaluate new compounds. Using “omic” as phenotype is not limited to coding gene transcription profiles associated with diseases. Further development of the TDS concept, adapting the *in silico* tools we have developed, may extend its application to any quantifiable disease trait. For example, non-coding RNA (ncRNA) has been discovered to be involved in cancer development, invasion and metastasis, as well as, drug resistance of melanoma^89,90^. Several studies have also provided convincing association between ncRNA expression and disease types^91–95^. Additionally, the exact model system we have implemented in this study (i.e., *xmrk* transgenic melanoma-bearing medaka) has previously been shown to exhibit piRNA profile changes between different melanoma stages^96^. These reports support the feasibility of adapting the TDS screening strategy to new and different disease biomarkers and phenotypes.

In summary, TDS drug screening strategy is capable of detecting transcriptional phenotypic changes induced by therapeutic drugs within fully intact fish that model melanoma. The establishment of the screening pipeline using transgenic melanoma medaka allows us to expand this strategy to other disease models for TDS screening pipeline development.

## Conclusion

We have established a novel phenotypic compound-screening pipeline. We conclude that using disease-related changes gene expression patterns, the transcriptional disease signature (TDS), as a first screen for compounds having drug potential is effective in terms of identifying transcriptional changes induced by two test compounds, while concurrently evaluating their off target toxicity.

## Electronic supplementary material

Supplementary Dataset

## References

[1] Herper, M. *The Cost Of Creating A New Drug Now $5 Billion*, *Pushing Big Pharma To Change*. Forbes, 2013.

[2] Lindsay MA (2003). Target discovery. Nat Rev Drug Discov.

[3] Munos B (2009). Lessons from 60 years of pharmaceutical innovation. Nat Rev Drug Discov.

[4] Silber, B. M. Driving drug discovery: the fundamental role of academic labs. *Sci Transl Med* **2**(30), p. 30cm16 (2010). 10.1126/scitranslmed.3000169 20445199

[5] Williams M (2005). Systems and integrative biology as alternative guises for pharmacology: prime time for an iPharm concept?. Biochem Pharmacol.

[6] Flordellis CS (2006). Rethinking target discovery in polygenic diseases. Curr Top Med Chem.

[7] Swinney DC, Anthony J (2011). How were new medicines discovered?. Nat Rev Drug Discov.

[8] Kokel D (2010). Rapid behavior-based identification of neuroactive small molecules in the zebrafish. Nat Chem Biol.

[9] White RM (2011). DHODH modulates transcriptional elongation in the neural crest and melanoma. Nature.

[10] Gut P (2013). Whole-organism screening for gluconeogenesis identifies activators of fasting metabolism. Nat Chem Biol.

[11] Ridges S (2012). Zebrafish screen identifies novel compound with selective toxicity against leukemia. Blood.

[12] Ni TT (2011). Discovering small molecules that promote cardiomyocyte generation by modulating Wnt signaling. Chem Biol.

[13] Zhong H, Lin S (2011). Chemical screening with zebrafish embryos. Methods Mol Biol.

[14] Hirota T (2010). High-throughput chemical screen identifies a novel potent modulator of cellular circadian rhythms and reveals CKIalpha as a clock regulatory kinase. PLoS Biol.

[15] Peal DS (2011). Novel chemical suppressors of long QT syndrome identified by an *in vivo* functional screen. Circulation.

[16] Clifton JD (2010). Identification of novel inhibitors of dietary lipid absorption using zebrafish. PLoS One.

[17] Sun Y (2012). Zebrafish chemical screening reveals the impairment of dopaminergic neuronal survival by cardiac glycosides. PLoS One.

[18] Wagner BK (2011). A small-molecule screening strategy to identify suppressors of statin myopathy. ACS Chem Biol.

[19] Das BC (2010). A forward chemical screen in zebrafish identifies a retinoic acid derivative with receptor specificity. PLoS One.

[20] Hao J (2010). *In vivo* structure-activity relationship study of dorsomorphin analogues identifies selective VEGF and BMP inhibitors. ACS Chem Biol.

[21] Molina G (2009). Zebrafish chemical screening reveals an inhibitor of Dusp6 that expands cardiac cell lineages. Nat Chem Biol.

[22] Kitambi SS (2009). Small molecule screen for compounds that affect vascular development in the zebrafish retina. Mech Dev.

[23] Ou HC (2009). Identification of FDA-approved drugs and bioactives that protect hair cells in the zebrafish (Danio rerio) lateral line and mouse (Mus musculus) utricle. J Assoc Res Otolaryngol.

[24] Hultman KA, Scott AW, Johnson SL (2008). Small molecule modifier screen for kit-dependent functions in zebrafish embryonic melanocytes. Zebrafish.

[25] Murphey RD (2006). A chemical genetic screen for cell cycle inhibitors in zebrafish embryos. Chem Biol Drug Des.

[26] Shafizadeh E, Peterson RT, Lin S (2004). Induction of reversible hemolytic anemia in living zebrafish using a novel small molecule. Comp Biochem Physiol C Toxicol Pharmacol.

[27] Nath AK (2013). Chemical and metabolomic screens identify novel biomarkers and antidotes for cyanide exposure. FASEB J.

[28] Baraban SC, Dinday MT, Hortopan GA (2013). Drug screening in Scn1a zebrafish mutant identifies clemizole as a potential Dravet syndrome treatment. Nat Commun.

[29] Jin S (2013). An *in vivo* zebrafish screen identifies organophosphate antidotes with diverse mechanisms of action. J Biomol Screen.

[30] Chen, J.R. *et al*. Live Fluorescent Staining Platform for Drug-Screening and Mechanism-Analysis in Zebrafish for Bone Mineralization. *Molecules* **22**(12) (2017). 10.3390/molecules22122068 PMC6149919 29186901

[31] Outtandy, P. *et al*. Zebrafish as a model for kidney function and disease. *Pediatr Nephrol* (2018). 10.1007/s00467-018-3921-7 PMC6424945 29502161

[32] Haggard DE (2018). Transcriptomic and phenotypic profiling in developing zebrafish exposed to thyroid hormone receptor agonists. Reprod Toxicol.

[33] Babu A (2018). Chemical and genetic rescue of an ep300 knockdown model for Rubinstein Taybi Syndrome in zebrafish. Biochim Biophys Acta.

[34] Gore, A.V. *et al*. The zebrafish: A fintastic model for hematopoietic development and disease. *Wiley Interdiscip Rev Dev Biol* (2018). 10.1002/wdev.312 PMC6785202 29436122

[35] Ibhazehiebo, K. *et al*. A novel metabolism-based phenotypic drug discovery platform in zebrafish uncovers HDACs 1 and 3 as a potential combined anti-seizure drug target. *Brain* (2018). 10.1093/brain/awx364 PMC5837409 29373639

[36] Eimon PM (2018). Brain activity patterns in high-throughput electrophysiology screen predict both drug efficacies and side effects. Nat Commun.

[37] Carreno Gutierrez H (2018). Automatic quantification of juvenile zebrafish aggression. J Neurosci Methods.

[38] Gerlai R, Fernandes Y, Pereira T (2009). Zebrafish (Danio rerio) responds to the animated image of a predator: towards the development of an automated aversive task. Behav Brain Res.

[39] McGown A, Shaw DP, Ramesh T (2016). ZNStress: a high-throughput drug screening protocol for identification of compounds modulating neuronal stress in the transgenic mutant sod1G93R zebrafish model of amyotrophic lateral sclerosis. Mol Neurodegener.

[40] Milan DJ (2009). Drug-sensitized zebrafish screen identifies multiple genes, including GINS3, as regulators of myocardial repolarization. Circulation.

[41] Patton EE, Nairn RS (2010). Xmrk in medaka: a new genetic melanoma model. J Invest Dermatol.

[42] Schartl M (2010). A mutated EGFR is sufficient to induce malignant melanoma with genetic background-dependent histopathologies. J Invest Dermatol.

[43] Wittbrodt J (1989). Novel putative receptor tyrosine kinase encoded by the melanoma-inducing Tu locus in Xiphophorus. Nature.

[44] Wellbrock C (2002). Activation of p59(Fyn) leads to melanocyte dedifferentiation by influencing MKP-1-regulated mitogen-activated protein kinase signaling. J Biol Chem.

[45] Morcinek JC (2002). Activation of STAT5 triggers proliferation and contributes to anti-apoptotic signalling mediated by the oncogenic Xmrk kinase. Oncogene.

[46] Wellbrock C, Schartl M (2000). Activation of phosphatidylinositol 3-kinase by a complex of p59fyn and the receptor tyrosine kinase Xmrk is involved in malignant transformation of pigment cells. Eur J Biochem.

[47] Geissinger E (2002). Autocrine stimulation by osteopontin contributes to antiapoptotic signalling of melanocytes in dermal collagen. Cancer Res.

[48] Wellbrock C, Schartl M (1999). Multiple binding sites in the growth factor receptor Xmrk mediate binding to p59fyn, GRB2 and Shc. Eur J Biochem.

[49] Schartl M, Walter RB (2016). Xiphophorus and Medaka Cancer Models. Adv Exp Med Biol.

[50] Lu, Y. *et al*. Comparison of Xiphophorus and human melanoma transcriptomes reveals conserved pathway interactions. Pigment Cell Melanoma Res (2018). 10.1111/pcmr.12686 PMC6013346 29316274

[51] Mishra RR, Kneitz S, Schartl M (2014). Comparative analysis of melanoma deregulated miRNAs in the medaka and Xiphophorus pigment cell cancer models. Comp Biochem Physiol C Toxicol Pharmacol.

[52] Klotz, B. *et al*. Expression signatures of early-stage and advanced medaka melanomas. *Comp Biochem Physiol C Toxicol Pharmacol* (2017). 10.1016/j.cbpc.2017.11.005 PMC5936653 29162497

[53] Lu Y (2015). Molecular genetic response of Xiphophorus maculatus-X. couchianus interspecies hybrid skin to UVB exposure. Comp Biochem Physiol C Toxicol Pharmacol.

[54] Boswell W (2015). Sex-specific molecular genetic response to UVB exposure in Xiphophorus maculatus skin. Comp Biochem Physiol C Toxicol Pharmacol.

[55] Chang J (2015). Molecular genetic response to varied wavelengths of light in Xiphophorus maculatus skin. Comp Biochem Physiol C Toxicol Pharmacol.

[56] Walter RB (2015). Exposure to fluorescent light triggers down regulation of genes involved with mitotic progression in Xiphophorus skin. Comp Biochem Physiol C Toxicol Pharmacol.

[57] Walter RB (2018). Waveband specific transcriptional control of select genetic pathways in vertebrate skin (Xiphophorus maculatus). BMC Genomics.

[58] Garcia TI (2012). Effects of short read quality and quantity on a de novo vertebrate transcriptome assembly. Comp Biochem Physiol C Toxicol Pharmacol.

[59] Kim D (2013). TopHat2: accurate alignment of transcriptomes in the presence of insertions, deletions and gene fusions. Genome Biol.

[60] Dobin A (2013). STAR: ultrafast universal RNA-seq aligner. Bioinformatics.

[61] Liao Y, Smyth GK, Shi W (2014). featureCounts: an efficient general purpose program for assigning sequence reads to genomic features. Bioinformatics.

[62] Li B, Dewey CN (2011). RSEM: accurate transcript quantification from RNA-Seq data with or without a reference genome. BMC Bioinformatics.

[63] Anders S (2013). Count-based differential expression analysis of RNA sequencing data using R and Bioconductor. Nat Protoc.

[64] Robinson MD, McCarthy DJ, Smyth GK (2010). edgeR: a Bioconductor package for differential expression analysis of digital gene expression data. Bioinformatics.

[65] Love MI, Huber W, Anders S (2014). Moderated estimation of fold change and dispersion for RNA-seq data with DESeq 2. Genome Biol.

[66] Lamb J (2006). The Connectivity Map: using gene-expression signatures to connect small molecules, genes, and disease. Science.

[67] Subramanian A (2005). Gene set enrichment analysis: a knowledge-based approach for interpreting genome-wide expression profiles. Proc Natl Acad Sci USA.

[68] Mootha VK (2003). PGC-1alpha-responsive genes involved in oxidative phosphorylation are coordinately downregulated in human diabetes. Nat Genet.

[69] Hollander, M., Douglas, A. S. & Chicken, E. *Nonparametric Statistical Methods* (1973).

[70] Geiss GK (2008). Direct multiplexed measurement of gene expression with color-coded probe pairs. Nat Biotechnol.

[71] Gilmartin AG (2011). GSK1120212 (JTP-74057) is an inhibitor of MEK activity and activation with favorable pharmacokinetic properties for sustained *in vivo* pathway inhibition. Clin Cancer Res.

[72] Flaherty KT (2012). Improved survival with MEK inhibition in BRAF-mutated melanoma. N Engl J Med.

[73] Megahed AI, Koon HB (2014). What is the role of chemotherapy in the treatment of melanoma?. Curr Treat Options Oncol.

[74] Barabas K (2008). Cisplatin: a review of toxicities and therapeutic applications. Vet Comp Oncol.

[75] Dunn LL (2006). The function of melanotransferrin: a role in melanoma cell proliferation and tumorigenesis. Carcinogenesis.

[76] Jin B (2016). Downregulation of betaine homocysteine methyltransferase (BHMT) in hepatocellular carcinoma associates with poor prognosis. Tumour Biol.

[77] Wang DG, Li TM, Liu X (2018). RHCG suppresses cervical cancer progression through inhibiting migration and inducing apoptosis regulated by TGF-beta1. Biochem Biophys Res Commun.

[78] Ming XY (2018). RHCG Suppresses Tumorigenicity and Metastasis in Esophageal Squamous Cell Carcinoma via Inhibiting NF-kappaB Signaling and MMP1 Expression. Theranostics.

[79] Strand SH (2017). RHCG and TCAF1 promoter hypermethylation predicts biochemical recurrence in prostate cancer patients treated by radical prostatectomy. Oncotarget.

[80] Iovanna JL (2017). Autophagy contributes to the initiation of pancreatic cancer. Med Sci (Paris).

[81] Quesada-Calvo F (2017). OLFM4, KNG1 and Sec 24C identified by proteomics and immunohistochemistry as potential markers of early colorectal cancer stages. Clin Proteomics.

[82] Mayama A (2018). OLFM4, LY6D and S100A7 as potent markers for distant metastasis in estrogen receptor-positive breast carcinoma. Cancer Sci.

[83] Hughes TR (2000). Functional discovery via a compendium of expression profiles. Cell.

[84] Ganter B (2005). Development of a large-scale chemogenomics database to improve drug candidate selection and to understand mechanisms of chemical toxicity and action. J Biotechnol.

[85] Schartl M (2015). Whole Body Melanoma Transcriptome Response in Medaka. PLoS One.

[86] Smith S, Grima R (2018). Single-cell variability in multicellular organisms. Nat Commun.

[87] de Haas EC (2010). Association of PAI-1 gene polymorphism with survival and chemotherapy-related vascular toxicity in testicular cancer. Cancer.

[88] Tukey RH, Strassburg CP (2000). Human UDP-glucuronosyltransferases: metabolism, expression, and disease. Annu Rev Pharmacol Toxicol.

[89] Romano G, Kwong LN (2017). miRNAs, Melanoma and Microenvironment: An Intricate Network. Int J Mol Sci.

[90] Fattore L (2017). MicroRNAs in melanoma development and resistance to target therapy. Oncotarget.

[91] Chen, X. *et al*. Predicting miRNA-disease association based on inductive matrix completion. *Bioinformatics* (2018). 10.1093/bioinformatics/bty503 29939227

[92] Chen X (2018). BNPMDA: Bipartite Network Projection for MiRNA-Disease Association prediction. Bioinformatics.

[93] Chen X, Yan GY (2013). Novel human lncRNA-disease association inference based on lncRNA expression profiles. Bioinformatics.

[94] Chen X (2018). MDHGI: Matrix Decomposition and Heterogeneous Graph Inference for miRNA-disease association prediction. PLoS Comput Biol.

[95] Chen X, Huang L (2017). LRSSLMDA: Laplacian Regularized Sparse Subspace Learning for MiRNA-Disease Association prediction. PLoS Comput Biol.

[96] Kneitz S (2016). Germ cell and tumor associated piRNAs in the medaka and Xiphophorus melanoma models. BMC Genomics.

