# Supplementary material for: Application of the Transcriptional Disease Signature (TDSs) to Screen Melanoma-Effective Compounds in a Small Fish Model
Source: Sci Rep. 2019 Jan 24;9:530. doi: 10.1038/s41598-018-36656-x (PMC6345854; doi:10.1038/s41598-018-36656-x)
Supplement: Supplementary file 1 — Supplementary Dataset [file 41598_2018_36656_MOESM1_ESM.pdf]

Application of the Transcriptional Disease Signature (TDSs) to Screen Melanoma-Effective Compounds in a Small Fish Model

Yuan Lu[1], William Boswell[1], Mikki Boswell[1], Barbara Klotz[2,3], Susanne Kneitz[2,3], Janine Regneri[2,3], Markita Savage[1], Cristina Mendoza[1], John Postlethwait[5], Wesley C Warren[6], Manfred Schartl[2,3,4], Ronald Walter[1]

[1]*Xiphophorus* Genetic Stock Center, Department of Chemistry and Biochemistry, 419 Centennial Hall, Texas State University, San Marcos, TX, USA. [2]Developmental Biochemistry, Biozentrum, University of Würzburg, Würzburg, Germany. [3]Comprehensive Cancer Center Mainfranken, University Clinic Würzburg, D-97074 Würzburg, Germany. [4]Hagler Institute for Advanced Studies and Department of Biology, Texas A&M University, College Station, USA. [5]Institute of Neuroscience, University of Oregon, Eugene, Oregon, USA. [6]University of Missouri, Columbia, Missouri, USA.

Supplementary Figures

Figure S1 Transcriptional Disease Signature (TDS) molecule screen overview

The TDS based molecule screening strategy serves as an extra filter between target based screening identifying lead compounds, and further mammalian animal model based preclinical studies. Small fish (e.g., medaka), are compatible with the well size of common 96-well-plate and thus commercially available drug libraries and screening technology can be applied for drug screen. Compounds can be administered to a fish-loaded 96 well plate manually or using robotic arm. Tissue homogenization is automated using a TissueLyser II that crushes the whole animal tissue by shaking with stainless beads (25 Hz frequency). RNA isolation is processed using QIAcube HT isolation station equipped with a programmable robotic arm. The robotic arm attaches to a 8-channel pipette capable of picking up and ejecting pipette tips, self cleaning and transfer of reagents between master reservoirs and the 96 sample wells. Gene expression is measured by direct mRNA molecule count using NanoString nCounter system that includes a automated preparation station and a scanner that quantify the signal incited by the Nanostring target-specific probes. The mRNA quantification results are downloaded and transferred to a custom data analyses pipeline for compound identification and scoring to find those that exhibited promising transcriptional effects for more detailed studies.

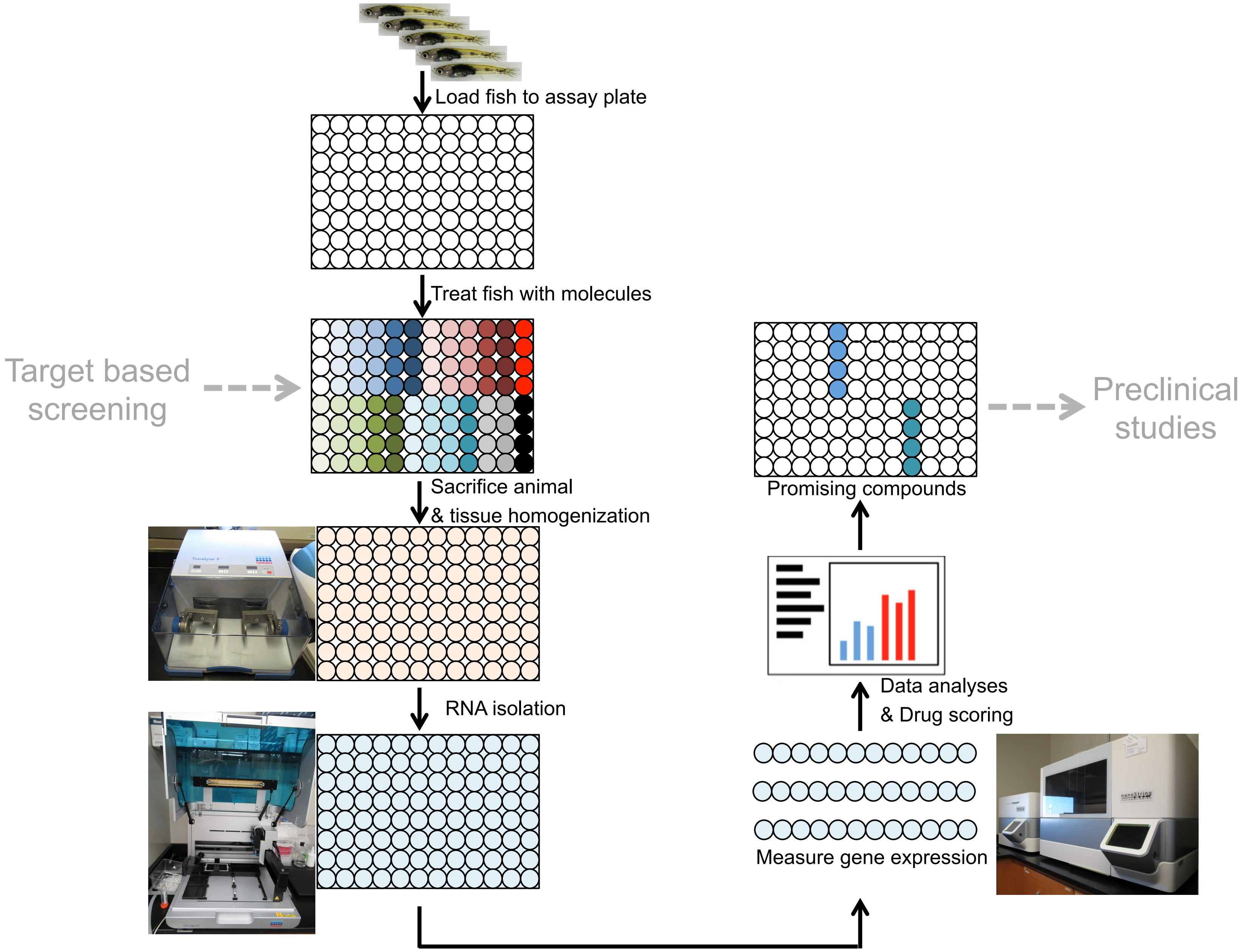
Load fish to assay plate
Treat fish with molecules
Target based screening
Sacrifice animal
& tissue homogenization
RNA isolation
Measure gene expression
Data analyses
& Drug scoring
Promising compounds
Preclinical studies

Supplementary Tables

Table S1 RNA-Seq statistics

Table S2 RNA-Seq differentially expression analyses results

Table S3 Nanostring nCounter panel

Table S1 RNA-Seq statistics

Sample name	Pre-filtered (In Million)	Post-filtered (In Million)	Mapping rate
WT_1	61.89	41.22	81.8%
WT_2	87.65	59.42	82.3%
WT_3	62.63	42.21	80.5%
WT_4	63.79	43.17	81.1%
WT_5	106.32	72.72	82.0%
WT_6	79.08	52.88	78.8%
WT_7	92.26	61.99	81.1%
WT_8	94.86	64.02	82.0%
WT_9	85.84	58.29	81.2%
WT_10	74.37	50.90	81.6%
Tg-mel_11	80.60	54.60	81.7%
Tg-mel_12	75.10	50.87	81.5%
Tg-mel_13	75.94	51.53	82.7%
Tg-mel_14	79.39	53.74	80.9%
Tg-mel_15	80.88	55.35	82.0%
Tg-mel_16	78.83	54.27	82.1%
Tg-mel_17	84.02	57.35	79.0%
Tg-mel_18	84.02	57.08	80.9%
Tg-mel_19	83.69	57.43	82.2%
Tg-mel_20	79.26	54.23	82.1%

Table S2 RNA-Seq differentially expression analyses results

Gene ID	Log_2FC (Tophat2-edgeR)	Log_2FC (rsem-DESeq2)	note (pass= kept in final panel, discarded= filtered out)
ENSORLG00000000443	1.93	0.59	pass
ENSORLG00000000597	1.63	1.03	pass
ENSORLG00000000675	1.90	1.66	pass
ENSORLG00000001248	-2.15	-1.97	pass
ENSORLG00000001307	-6.87	-5.90	pass
ENSORLG00000002102	0.28	1.03	pass
ENSORLG00000002182	1.23	1.14	pass
ENSORLG00000002315	-2.54	-2.80	pass
ENSORLG00000002431	-1.34	-1.39	pass
ENSORLG00000002478	1.60	1.43	pass
ENSORLG00000002484	1.63	1.60	pass
ENSORLG00000002540	-1.37	-1.16	pass
ENSORLG00000002555	-0.82	-1.87	pass
ENSORLG00000002629	-1.78	-0.69	pass
ENSORLG00000002760	1.41	1.38	pass
ENSORLG00000003027	1.06	1.10	pass
ENSORLG00000003119	1.00	1.03	pass
ENSORLG00000003410	1.51	1.42	pass
ENSORLG00000003976	-1.03	-1.01	pass
ENSORLG00000003976.2	-1.03	-1.01	pass
ENSORLG00000004118	-1.94	-1.84	pass
ENSORLG00000004326	1.53	1.58	pass
ENSORLG00000004344	1.34	1.31	pass
ENSORLG00000004413	-1.53	-1.69	pass
ENSORLG00000004452	1.82	1.96	pass
ENSORLG00000004539	1.77	1.78	pass
ENSORLG00000004723	1.27	1.27	pass
ENSORLG00000004774	1.11	1.03	pass
ENSORLG00000005218	-4.87	-2.93	pass
ENSORLG00000005485	-1.21	-0.53	pass
ENSORLG00000005563	-1.23	-1.24	pass
ENSORLG00000005947	4.48	3.68	pass
ENSORLG00000006038	1.58	1.61	pass
ENSORLG00000006368	1.28	1.24	pass
ENSORLG00000006812	0.98	1.18	pass
ENSORLG00000007024	-1.67	-1.96	pass
ENSORLG00000007074	1.91	2.04	pass
ENSORLG00000007163	1.31	1.36	pass
ENSORLG00000007497	1.69	1.68	pass
ENSORLG00000007808	1.33	1.15	pass
ENSORLG00000007864	2.13	2.16	pass
ENSORLG00000008054	1.20	1.24	pass
ENSORLG00000008103	0.95	1.05	pass

ENSORLG00000008285	1.03	1.23	pass
ENSORLG00000008290	2.64	2.57	pass
ENSORLG00000008473	2.59	2.53	pass
ENSORLG00000009400	1.40	1.38	pass
ENSORLG00000010154	-2.93	-1.73	pass
ENSORLG00000011733	1.08	0.74	pass
ENSORLG00000011903	3.24	3.59	pass
ENSORLG00000011996	-1.07	-0.98	pass
ENSORLG00000012209	1.48	2.12	pass
ENSORLG00000012783	1.97	2.03	pass
ENSORLG00000012958	0.78	1.18	pass
ENSORLG00000012977	2.01	2.00	pass
ENSORLG00000013017	1.08	1.17	pass
ENSORLG00000013074	1.46	1.39	pass
ENSORLG00000013461	1.15	1.18	pass
ENSORLG00000013629	0.99	0.93	pass
ENSORLG00000013692	-1.91	-1.54	pass
ENSORLG00000013773	-1.29	-0.66	pass
ENSORLG00000014007	1.44	1.29	pass
ENSORLG00000014358	-1.26	-1.28	pass
ENSORLG00000014802	1.31	1.14	pass
ENSORLG00000014854	0.86	1.10	pass
ENSORLG00000015001	1.83	1.85	pass
ENSORLG00000015618	1.35	1.17	pass
ENSORLG00000015645	-1.36	-0.94	pass
ENSORLG00000015663	-1.21	-0.13	pass
ENSORLG00000015690	-5.56	-4.82	pass
ENSORLG00000015785	0.95	1.01	pass
ENSORLG00000015824	1.07	1.05	pass
ENSORLG00000015952	-1.03	-1.01	pass
ENSORLG00000016829	-1.31	-0.96	pass
ENSORLG00000016946	1.07	1.01	pass
ENSORLG00000017109	2.23	2.06	pass
ENSORLG00000017153	-2.74	-4.96	pass
ENSORLG00000017411	-2.35	-2.41	pass
ENSORLG00000017942	-2.38	-2.38	pass
ENSORLG00000018272	1.31	1.25	pass
ENSORLG00000018315	-2.15	-2.14	pass
ENSORLG00000018856	-0.82	-1.14	pass
ENSORLG00000018864	-3.55	-1.89	pass
ENSORLG00000019168	-1.09	-1.16	pass
ENSORLG00000019328	1.26	1.23	pass
ENSORLG00000019336	1.19	1.19	pass
ENSORLG00000019355	-7.96	-6.73	pass
ENSORLG00000019589	3.00	2.89	pass
ENSORLG00000019686	-1.59	-1.74	pass

ENSORLG00000019793	0.19	-1.39	pass
ENSORLG00000019887	-10.91	-6.25	pass
ENSORLG00000019909	1.40	1.44	pass
ENSORLG00000019947	-2.89	-3.25	pass
ENSORLG00000020013	-2.25	-1.43	pass
ENSORLG00000020643	1.00	1.02	pass
ENSORLG00000020751	-1.33	-0.93	pass
Xmrk	na	na	pass
ENSORLG00000000026	5.98	5.42	discarded
ENSORLG00000000313	-1.16	-1.19	discarded
ENSORLG00000000491	1.76	1.97	discarded
ENSORLG00000000504	-1.05	-1.16	discarded
ENSORLG00000000605	2.67	1.13	discarded
ENSORLG00000001003	2.28	1.96	discarded
ENSORLG00000001131	-1.23	-1.22	discarded
ENSORLG00000001187	-0.55	-3.49	discarded
ENSORLG00000002163	-2.75	-2.49	discarded
ENSORLG00000002303	-1.81	-0.95	discarded
ENSORLG00000002401	-1.54	-1.20	discarded
ENSORLG00000002649	-1.00	-1.13	discarded
ENSORLG00000002652	3.57	3.31	discarded
ENSORLG00000003041	-0.50	-1.08	discarded
ENSORLG00000003101	-1.31	-1.31	discarded
ENSORLG00000003103	-1.32	-1.30	discarded
ENSORLG00000003123	1.59	1.54	discarded
ENSORLG00000003451	1.24	1.12	discarded
ENSORLG00000003571	1.36	1.49	discarded
ENSORLG00000004187	0.80	2.50	discarded
ENSORLG00000004282	-1.14	-1.14	discarded
ENSORLG00000005073	-1.91	-1.89	discarded
ENSORLG00000005199	-1.09	-1.24	discarded
ENSORLG00000005316	1.60	1.58	discarded
ENSORLG00000005473	1.50	1.00	discarded
ENSORLG00000005506	1.46	1.49	discarded
ENSORLG00000005588	1.04	1.05	discarded
ENSORLG00000006046	0.99	0.86	discarded
ENSORLG00000006142	-1.04	-0.48	discarded
ENSORLG00000006224	1.70	1.59	discarded
ENSORLG00000006302	2.07	2.20	discarded
ENSORLG00000006451	1.17	1.00	discarded
ENSORLG00000006798	1.84	0.01	discarded
ENSORLG00000006976	-1.06	-1.15	discarded
ENSORLG00000007232	2.33	2.45	discarded
ENSORLG00000007559	1.20	1.27	discarded
ENSORLG00000007765	0.94	1.06	discarded
ENSORLG00000007849	-1.39	-1.38	discarded

ENSORLG00000007901	-1.23	-1.04	discarded
ENSORLG00000007929	2.66	2.85	discarded
ENSORLG00000007987	-1.08	-1.17	discarded
ENSORLG00000008028	-1.20	-1.06	discarded
ENSORLG00000008060	0.97	1.00	discarded
ENSORLG00000008113	-1.30	-1.27	discarded
ENSORLG00000008184	-1.26	-1.25	discarded
ENSORLG00000008419	0.99	0.98	discarded
ENSORLG00000008608	-1.02	-0.34	discarded
ENSORLG00000008701	-0.83	-1.02	discarded
ENSORLG00000008715	-1.33	-1.33	discarded
ENSORLG00000009164	-1.64	-1.43	discarded
ENSORLG00000009169	-1.25	-1.15	discarded
ENSORLG00000009262	3.10	3.31	discarded
ENSORLG00000009502	-1.28	-1.02	discarded
ENSORLG00000009880	-3.76	-1.79	discarded
ENSORLG00000009925	1.95	1.32	discarded
ENSORLG00000010038	1.69	1.66	discarded
ENSORLG00000010054	-2.64	-0.90	discarded
ENSORLG00000010177	-4.81	-3.66	discarded
ENSORLG00000010256	1.11	1.04	discarded
ENSORLG00000010277	0.92	0.93	discarded
ENSORLG00000010656	-1.02	-1.06	discarded
ENSORLG00000010880	-2.47	-2.56	discarded
ENSORLG00000010905	1.31	1.33	discarded
ENSORLG00000011293	2.47	2.52	discarded
ENSORLG00000011373	-2.64	-3.01	discarded
ENSORLG00000011569	1.22	1.20	discarded
ENSORLG00000011826	-1.26	-0.86	discarded
ENSORLG00000012026	1.64	1.66	discarded
ENSORLG00000012171	-1.23	-1.02	discarded
ENSORLG00000012352	1.02	0.60	discarded
ENSORLG00000012423	-1.09	-0.67	discarded
ENSORLG00000012679	0.99	0.70	discarded
ENSORLG00000012848	1.48	1.55	discarded
ENSORLG00000012895	-1.71	-1.77	discarded
ENSORLG00000013345	1.66	1.51	discarded
ENSORLG00000013456	1.62	1.63	discarded
ENSORLG00000013462	1.63	1.62	discarded
ENSORLG00000013468	-1.35	-1.07	discarded
ENSORLG00000013560	1.24	1.17	discarded
ENSORLG00000014572	-1.30	-1.51	discarded
ENSORLG00000014926	-1.46	-1.39	discarded
ENSORLG00000015051	2.82	2.78	discarded
ENSORLG00000015052	3.66	3.69	discarded
ENSORLG00000015055	2.62	2.59	discarded

ENSORLG00000015345	-1.24	-1.12	discarded
ENSORLG00000015462	-1.03	-1.12	discarded
ENSORLG00000015481	1.48	1.69	discarded
ENSORLG00000015893	1.25	1.24	discarded
ENSORLG00000015951	1.67	1.66	discarded
ENSORLG00000016164	-3.98	-0.19	discarded
ENSORLG00000017099	-1.17	-1.28	discarded
ENSORLG00000017168	0.31	1.05	discarded
ENSORLG00000017172	-1.58	-0.84	discarded
ENSORLG00000017233	1.02	1.12	discarded
ENSORLG00000017257	-1.20	-1.10	discarded
ENSORLG00000017658	1.51	1.51	discarded
ENSORLG00000017946	-1.82	-1.99	discarded
ENSORLG00000018160	-2.28	-3.27	discarded
ENSORLG00000018261	2.39	0.12	discarded
ENSORLG00000018417	-1.86	-0.52	discarded
ENSORLG00000018470	-1.11	-1.51	discarded
ENSORLG00000018566	1.13	1.27	discarded
ENSORLG00000018575	1.35	1.33	discarded
ENSORLG00000018596	1.18	1.04	discarded
ENSORLG00000018598	-1.28	-1.13	discarded
ENSORLG00000019009	-1.86	-1.98	discarded
ENSORLG00000019174	-1.40	-1.46	discarded
ENSORLG00000019182	-1.06	-1.23	discarded
ENSORLG00000019338	-6.02	-4.06	discarded
ENSORLG00000019564	2.71	4.19	discarded
ENSORLG00000019576	2.82	2.81	discarded
ENSORLG00000019728	-0.02	-17.75	discarded
ENSORLG00000019792	-1.70	-1.39	discarded
ENSORLG00000019881	1.83	1.09	discarded
ENSORLG00000020085	-3.62	-11.82	discarded
ENSORLG00000020164	0.52	-2.83	discarded
ENSORLG00000020198	-0.42	-1.10	discarded
ENSORLG00000020268	-2.28	-1.04	discarded
ENSORLG00000020303	-1.39	-1.30	discarded
ENSORLG00000020370	-0.66	-1.76	discarded
ENSORLG00000020430	1.58	-3.26	discarded
ENSORLG00000020464	-3.77	-1.11	discarded
ENSORLG00000020473	-0.88	-1.10	discarded
ENSORLG00000020621	-1.02	-1.04	discarded
ENSORLG00000020767	0.37	1.47	discarded

Table S3 Nanostring nCounter panel

probe.name	weight	gene.name	logFC	probe.type	signaling/func	Target Sequence
ENSORLG00000000443	0.91	gstr	0.66	tds	tumor	CCAGAAGAACAAGCTCTGATGTACCAGCGCATGTTTGAGGGCCTCACGTTCTACGAGAAGCTCGGAAGCGTCATCTACTACGATTGGTACGTCCCTGAAG
ENSORLG00000000597	0.73	uncharacteirzed.5	0.51	tds	tumor	ATCGCGACCACAGCCCTCGCCGTCGAGAGAGTGACGATGAAGGAGACAGGGAAGGTGGTGACCATAATGGAAAACATGGCCACAAGCATGGACATGGTCA
ENSORLG00000000675	0.94	ankrd34ba	1.37	tds	tumor	AGTCCACTCTTCCTCCAGTCTTTTGGGCGGTAAAAAGCTGGACACTGCTCCTCACGCCTCATCAAGTCTTTGTAGTGTAGTCCAGAAGCGTCAGCTTGGA
ENSORLG00000001248	2.00	trim14	-1.71	tds	tumor	TAACAACGATTTGGGCGACTTGGGACTGAAGCATCTTTGTTTTGGACTGGAGAACCCCAACTGTACTTTGGAAACTCTCAGTCTGTCAGGATGTCTGATC
ENSORLG00000001307	2.00	myo	-3.51	tds	tumor	AGCAGAACAAAGACGCGGATCCGATTCTGTGAAAGCCGTCCGCAAGCTCGAGCGCAGAATCAAGGAGCTCGTCTATCAGGAAGAACGCATGAGGCTGCAG
ENSORLG00000002102	0.70	lye	0.28	tds	tumor	ACACTAACTCATGTTCAGCCACAACTCAGGGGTTGACATCGTGGAAGTTTATCAATGGGATGTTTCAGGGAAACATTGAGCCTGTAAATGTACTGCGATG
ENSORLG00000002182	0.86	ppt2	0.61	tds	tumor	CTGGGCTGGGGGGTCCGCATACAAGCCGGTGATTATAGTCCACGGTTTGTTCGACAGCCCCAAAAATTTACTCAACTTGCAGCGATTCATCAGCGAGTCT
ENSORLG00000002315	0.94	ccdc109b	-1.32	tds	tumor	TTGCCTGGGACGTGATGGAACCCGTCACCTTTTTCATTTCCTGCACGACAAGCATGATCTTCTTTGCGTACTACATCCTCACCAAGCAGGACATCATCTT
ENSORLG00000002431	0.94	bhmt	-1.01	tds	tumor	GCTGTGAAGGCCATCTTCAAGAAGCAGATCGACGTCTTCATCAGGAAGGACGTGGACTTTCTGATCGCAGAGTACTTTGAGCACGTGGAGGAAGCAGAGT
ENSORLG00000002478	0.91	bscl2	0.74	tds	tumor	CCACAGATGGGGGAAACTCCAAGCAGCCAGGCGGAGCTACAAAAGGATTTCTCTACAGGTACTGCAGAGAACCTGGGTCCTGTGATAAAGGACAGTACAA
ENSORLG00000002484	0.84	bscl2l	0.70	tds	tumor	ATCCTGCTGAAGCTGCAGGATGCTGCTGCCATGGCGATCTCACGGGTTCGTGAGAGACTCGCACAGGGCCTGACTGTCCTCTCTGTTGTTCTTCTGCTAC
ENSORLG00000002540	0.92	tlr2	0.97	tds	tumor	GGTTTCCGGATGGAAACCTCTTTCCCAATCTGCAAACACTTGCTATACAGTCCAACACCCTGAACTTGTTTGGTCGTTCGGACCTCCAGTCATACAGACG
ENSORLG00000002555	2.00	uncharacteirzed.8	-2.09	tds	tumor	TCTTCTGTGAAACACAAAGACACATTGCACACCTCCTCTCCCCTGGACCCTGGAGGGCACTTGGCAGAAATGATATTCCGGGTCTGGCGAAGCAAGGGTG
ENSORLG00000002629	2.00	uncharacteirzed.9	-1.27	tds	tumor	CCCCTGATCCTCCTCACATCATATGGATCAGAATCACTGCTGGAGTGATGGTCTTCGTCCTACACATCACGGTCTTCCTCCTGTGCCTGTACACCTACCG
ENSORLG00000002760	0.70	slc24a5	0.44	tds	tumor	TTCAGGGACTGCCTGGCATATGGAATCAGTGTTGCCGCTGTTATTGGCATCATTTCCGATAACAAAGTGTACTGGTACGAGGCTGCCTGTCTGCTTCTAG
ENSORLG00000003027	0.85	adra2b	0.35	tds	tumor	TCGTGCACCTGTGTGCCATCTCTTTAGACCGTTACCTGTCAATCTCCAGGGTTACTTACGGACGTCAACGGACTCCCAGACGCATCAAAGCTGCCATTGT
ENSORLG00000003119	0.77	rab34b	0.40	tds	tumor	CCAGGGCGTTGAGCTTTAATGTGGCAAAGGTCATCGTGGTGGGGGATGTTGCCGTTGGGAAGACGTGTCTGATCAGCAGGTTCTGTAAGGGTGTATTTGA
ENSORLG00000003410	0.85	mreg	0.66	tds	tumor	CGCATGGGATCCACGGGGTACCGTCGTCTGAGTGTTGACATAGAGGCCATGAGAGATGTACGCAGAGAAGTCCGAGACAAATGGAAAACCATTCTGGAGA
ENSORLG00000003976	0.89	cyp3a30	-0.76	tds	tumor	AAATGAACCCATTGACATCAAACAGTTCGTGGCACCGTACAGTTTGGATGTTGTGACCAGCGCCTCTTTCAGCGTGGAAACAGACTGCATAAATAATCCA
ENSORLG00000003976.2	0.89	cyp3a30.2	-0.79	tds	tumor	CCACTCTGTCTCACATTTTCTACAACCTGGCCATAAACCCGGATGCAATGCACACCCTGCAGGAAGAAATTGATGCCAACCTACAAAAAGATGTTCCAGT
ENSORLG00000004118	2.00	stradb	-2.07	tds	tumor	CCTCATGGACTATGGATCGGCGGACACTTTACTGCGAGCACATTTCCCAGATGGAATGAGTGAATCCCTGATAGCTTATCTGCTGCACGGAGTACTGAAA
ENSORLG00000004326	0.74	tyrp1a	0.54	tds	tumor	GGCCCGGCGGCGACAGCCCGCAGTTCGAAAACGTCACCATTTACAACTACTTCGTTTGGACTCACTACTACTCCGTCAGCAAGACCTTCCTGGGAGCGGG
ENSORLG00000004344	0.88	kif5bb	0.76	tds	tumor	CCTGCAGGACTCTCTAGGAGGAAACTGCAGGACGACCATGGTGATCTGCTGCTCCCCGTCCTCCTTCAACGATGCAGAGACCAGGTCCACACTGATGTTT
ENSORLG00000004413	0.74	gckr	-1.07	tds	tumor	ACACTGTTCTGCTTATCTATTCTCAATCTGACGATCTCCATGAGGTGCTGAACTTAGGGAGAAGAGTGAGAGAAAAGACACCTAACCTCCACGCTGTTCA
ENSORLG00000004452	0.70	mast.cell.protease.1A	0.47	tds	tumor	GTGGGCATTGTCTCTTTTAACAAAAACAACAACTGCACATACCCTGATGTGCCCAACGTCTACACGGACATCAGAGAATATGTTGACTGGATCAAATACA
ENSORLG00000004539	0.82	tyr	0.55	tds	tumor	CCTTCTCTGGGTTGGACGACCGTGAAAAATGGCCCTTAGTTTTCTTCAACAGAACTTGTCAGTGCACAGGAAACTTCATGGGCTTCAATTGTGCAGATTG
ENSORLG00000004723	0.89	tyrp1b	0.41	tds	tumor	CAGGTCAGCTTCGGAGGCGTCGATTTCTCCCACGAGGGCCCGGGTTTTGTCACCTGGCACCGTTTTCATCTGCTGCAACTGGAGAGAGACATGCAGAACA
ENSORLG00000004774	0.97	dclre1a	0.64	tds	tumor	AGTCCTGAGTACACCTTTCCCAAACAGCAAGAGGTCATCAACTTTGCAGCCAACAGAGCCTTTGAGTCGGTCACCCTGAATCCCCGCACACTTGTGGTGT
ENSORLG00000005218	0.86	lei.2	-2.36	tds	tumor	GCAGCTGCTACAAAGCAGAACTGGAGTCTGTGGACTTTAAAAACAAATACGAGGAAGCCAGGATCAAGATCAACAGGGTGTCTTCAATGTTCTTACCAGG
ENSORLG00000005485	0.72	ubtf	-0.33	tds	tumor	TCTTCAGAAGTTGTTCGCTTCGTTAAAAGCCAACATCCCAGCAGAGGAGGAAGCTTTCATTTACACCAAAGGACTGAAACAAGTCGACTGGGAGAACGTC
ENSORLG00000005563	0.87	cuzd1.2	-0.88	tds	tumor	GTTGGGAAGGGTCTGCAACAACAACACCAGCTTGAGCGCTTTCTTTTCCACCTCCAACTACATGACCGTCGTTTTCCAGACCGACGGCTCTGTGGTCGCC
ENSORLG00000005947	0.89	chst12	0.71	tds	tumor	AACACGGAATCATCTACTGTTATATTCCTAAGGTGGCATGTACCAACTGGAAGAGGACCTTGATCGCTTTAAACTTTAGTGAACCATATCCTGACCCTAC
ENSORLG00000006038	0.92	gpr61	0.59	tds	tumor	CAACTCAGGAGACTTCTGAAGGTCTAGTTTCCACTCAAAGACATTGTTCCCTTCACTGGACAGGAAGCACAACCACACGCCTGCTTTTCATGGTCTTCTT
ENSORLG00000006368	0.81	plaur	0.57	tds	tumor	TTCAAAATCTCATTCGTGATTGAATGCTGCAACACAGACCTCTGCAACACCAAAGATGCAAGCGCGCCCAGCAAATCTATTCCAAATAAAATGAAGTGCT
ENSORLG00000006812	0.84	zrc1	0.80	tds	tumor	CTGGCTTTCGAGTTCATAGGTGTGGTGACCTTTTGTCAGGGGTCTCAAAGGTGTTGAAGGGTGTAGGAGTGAGCTGCTGCACTGTTCAGCCGGAGTTCAT
ENSORLG00000007024	2.00	g6pd	-0.85	tds	tumor	AACCTCCTGCACACCAACTTTTGCCACAGCAGTGCTCAATGTCCAGAATGAAAGATGGGATGGGGTTCCATTTATTCTGCGCTGCGGTAAAGCTCTAAAT
ENSORLG00000007074	0.93	uncharacteirzed.15	0.88	tds	tumor	TCAGGCAGCTGGATCTGGCTGACACCAAATCCATTTGCCTTTTTGCCGAGAGTATTTATAGCACGGAGAAAGCTCTTCACTACCTTGTCAACAATGCAGG
ENSORLG00000007163	0.72	mchr2	0.47	tds	tumor	CCTCTATCACGTTTTCCGGTCCATCCGGCGAGTCAAGCGAAAGCAGTCTGTGTGGGCGAAACGAGCCACTAAGATGGTCCTCATGGTTATCGCCGTGTTT
ENSORLG00000007497	0.85	tspan10	0.54	tds	tumor	CGCCATCCGAGAGAACTCCTGCTTACTGAGGCTCTTTTCAGGCATCTTGTTGATCCTCATCACAGTTCAGGTGTTGATCGCCATCATGGCCTACAGTATG
ENSORLG00000007808	0.92	tmc6a	0.68	tds	tumor	CAGGAACTTGTTGCTGAAGACGAGTCTTATTGGAGTGCTGTGTTACCGCTGGCTGTGGAGAATTGCTGAAATTCAACAATTTAAGTGCTGGGAGAGCTTT
ENSORLG00000007864	0.71	opn4xa	0.99	tds	tumor	TGGCCATCTCTATTGACCGCTACATAGTTATAACCAAGCCTCTGCAGTCCATAGCTTGGACTTCTGCAAGGCGCACTTGCTTTATTATTGCCCTGGTCTG
ENSORLG00000008054	0.70	slc2a11b	0.60	tds	tumor	TTTATCTTGAGTTTTGGCCTCGGACCAGGTGGAGTGACTAACATCCTCACCACAGAGCTGTTCACACAATCCTCACGCCCTGCAGCGTTCATCATCTCAG
ENSORLG00000008103	2.00	cep72	0.85	tds	tumor	CTTATATCGTGCACGCCTTGCCCAGCTTGCGAAAGCTTGACAGCTGCTCGGTCAGAGAGACGGAACGCAAAGTTGCCATCATGCAGTTCTCCTCTGATCT
ENSORLG00000008285	0.71	trpc2b	1.02	tds	tumor	AGATAAATTTGCCTCTAGACACCAGGACATTCTGCACAGCTCATTCCACATGGTGTGGGTGGTCGGATTCTTCTGGTATGAGTGTAAAGAAGTGTGGATT
ENSORLG00000008290	0.95	cutc	3.54	tds	tumor	GAGCTGTGTGCAAGCCTTCTGGAGGGAGGACTCACGCCCAGTCTAGGTTTGGTTCAGGTTGTGAAGCAGTATATCAAAATCCCGATCTTTGTGATGATCC

ENSORLG00000008473	0.90	PNPLA1	1.78	tds	tumor	GCAGTGACCCGTGTGACTGATGGGAAACAGTTGATACTATCCGATTTCAATTCCAAAGAAGATATAGTGCAGGCTTTGCTTTGCAGCTGCTTTGTGCCTG
ENSORLG00000009400	0.84	mc1r	0.71	tds	tumor	TGGACAACGTCATCGACGTGATGATCTGCAGCTCCGTGGTGTCATCGCTGTCTTTCCTGTGCACCATCGCCGCGGATCGCTACATCACCATCTTTTACGC
ENSORLG00000010154	0.75	cd62l	-2.16	tds	tumor	TGACCTGGGTTGATGATTACCAGTACTGCACCATGGACCAACGTTTTATCTGCTACAATGAAACTAATCCAACTCAGAAATTCCATCTAATAGAGACGCC
ENSORLG00000011733	0.84	rhcgb	0.48	tds	tumor	GCCTTATGGATCCCTGATTGTTGGGTTCTGCTGTGGAATTTTGTCCACACTTGGATACATCTACATCACGCCCTTCTTGGAAAAGTACCTGAAAATCCAG
ENSORLG00000011903	0.83	igh	0.89	tds	tumor	TGAACACAGGAAACAACTCTCCCACATATTCCAGTTCTTTTCAGGGTCGTTTCCTCATGACTGAAGTTGTGTCCAGCAGCTCTCAGTACCTCGAGATCAG
ENSORLG00000011996	0.70	ehf	0.46	tds	tumor	AAAGAGATCCTCCTGGGGAGGTATTAATCTACACTTCAGCAGAGGTTCAACCGGGGGTGGAAAACACAATAATTTGCTTTGTGAATGGTTTCTACCCACC
ENSORLG00000012209	0.91	vmp1	0.99	tds	tumor	ATGTGGCTCTGTGGATTTCCCAGAGCCTCCCTATCCGGACCAAATCGTGTGTCCCCAACAGGAAGTCACAACGGGGAGCATCTCTGTTTGGTCCATCATT
ENSORLG00000012783	0.81	pim2	1.69	tds	tumor	AACACCAGGATGTTCGGGATGAATATGCTGAGGATCAGCAAAGACCTGTCCCCGGACTGCCAGTGTTTTTTGAAATCGTGTCTGAGGTCTTACCCTTCAT
ENSORLG00000012958	2.00	il17d	1.15	tds	tumor	TCCCTGAGGCCTACTGTCTGTGCAAAGGCTGCTTGATTGGACCATCAGGAGAGGAGAGCAGGCAGTACCGCAGTACTCCAGTTTACTCTCCGTCCGTCAT
ENSORLG00000012977	2.00	uncharacteirzed.17	1.77	tds	tumor	TTCTGCTCCGATGTGGTGTGCAAATATTGGCCATATCTGCAGAAGGTTGTGCGCCACTGTCCTGAACTACAGGGGTTGTTGACAATGCATCCTTTCCTCT
ENSORLG00000013017	2.00	ift88	1.17	tds	tumor	ATTACGACCAAGCTGATCGATACGCAGATCTTGCTATGAACGCTAACCGCTACAACCCTGCTGCTCTCGTCAATAAGGGAAATACAGTGTTTGTCAAGCA
ENSORLG00000013074	0.89	prtfdc1	0.49	tds	tumor	TTTGAAATTCCCAACCGATTCGTCGTGGGTTATGCCCTGGACTACAATGAATACTTCCGTGACCTGAATCACATCTGTGTCATCAGTAAAACTGGAAAGC
ENSORLG00000013461	0.90	mitfb	0.34	tds	tumor	AGTTACAGTGATGACATCCTTGGTCTGATGGACCCAGGACTTCAGATGCCAAATACGATCCCTGTGCCTGCAAACCTCATGGACATGTACGGTAACCAGG
ENSORLG00000013629	0.88	ovgp1	1.11	tds	tumor	GATGACCTATGATTTCCATGGCACCTGGGAAAGTGTTACAGGACATTACAGCCCTTTATTCAAGGGATCTCGGGACTTTGGGGACCATGTCTACCATAAC
ENSORLG00000013692	2.00	p3h4	-3.89	tds	tumor	CTTTACCATTACCTCCAATACGCCTACTACAAATTGAATGACGGACGCAGAGCTGTGCCCTGTGCGTACAGCTATTTCCTGTTTAAGCCAGAGGACGACG
ENSORLG00000013773	0.76	nt5c3b	-0.41	tds	tumor	AACACATATTATCCAATAGAAATTGATGCGAGCCGAAGTGCAAAGGAGAAGCTGCCTTTCATGGTGGAATGGTGGACTAAAGTCCATGAGCTCCTGATCG
ENSORLG00000014007	0.96	zdhhc18b	0.68	tds	tumor	GCTTGTTCTTCATCTTCGACTGTCCCTTCCTGGTCAAACACCTGAGCAGCTGCGTTCCGGCTGTTGGAGCCGTGCTCTTCGTCTTCGTCTTCATCACGCT
ENSORLG00000014358	0.83	mep1b	-0.62	tds	tumor	CAATAGCGCAGCAGCTGATGATGTTCTTAACATTTGGGTGAAAGAGTATGACGTAAATAACTCTGGGGAGGAACTGAAGCTTTTCAAGAGCATTTCGGGG
ENSORLG00000014802	0.84	uncharacteirzed.19	0.52	tds	tumor	CCTCGGCGGACTGCTAGGCGTCCTGGACGACGTGGTTGTGGTCTTCCTGCTGCTCGTCTGCGTCATCAATGTCAACCAGCAAATTGTGCCTGATGAGGGA
ENSORLG00000014854	0.82	cracr2ab	0.36	tds	tumor	AGTCTGGGAGCCATTAATGTCTTCGAGGATCCAGGTGAGGTACAGAATCTTTGGACCCAGTTCAGACGAGATGAGCCACACCTTTTGTCCAATTTTGAGG
ENSORLG00000015001	2.00	pmela	0.94	tds	tumor	GGTGTCCAGTGTTGTGCCCATAGCCACCGCTTTGGATCAGAATGCTGTGGATGTCACCATTAGCTGTCAGGGAAGTCTTCCGAGTGAGGTCTGCACTATT
ENSORLG00000015618	0.99	g2e3	1.07	tds	tumor	TCTACAATGCAAATGCTGTCAGGGAGGATGCCTACTACATAGCTGGGAAGATGATTGCTGTGTCAGTTGTGCACGGTGGTCCGGGACCTCACTTCCTGTC
ENSORLG00000015645	0.96	rrnad1	-0.42	tds	tumor	GTTTTGACAAGCAGGGAGATTCTCATGGACCTTCGGACTGTTATGACTACCAGGAATTAAGCACAGCTCAAGGGGCGGAGTCATCTTCTGGTAACTGTTG
ENSORLG00000015663	0.93	cd83	0.70	tds	tumor	ACAGGGAAGGGAGGATTCTTCTCTCTGTGGAAGATTGTCCTACTGATACTGTGATGGAAACTGTCATAAGGGATACCAGCTTGGTTTTGTTTGCCACAGC
ENSORLG00000015690	0.88	cyp2w1	-3.36	tds	tumor	CAAGGGGTTTTTCATTGAGAAGGGCACAACAGTGATTCCAGTCTTGACGTCTGTCCTTTATGATGAGAATGAGTGGGAGAAGCCAAACATCTTCTATCCC
ENSORLG00000015785	0.84	fam177b	0.66	tds	tumor	ATCAAAGCCAATCCCCTTTGAGGAACCAGCAGGGAGGACCAAATTATCTTTCAAGAATGCGGCCCTTCTAGTAGGTAGGATTTCACTTCTAGCTTGCGAT
ENSORLG00000015824	2.00	lysmd1	0.93	tds	tumor	CTGTTCTGTCAGACCTGAAAGGGATTCATTTGTCTGAGGACTGTGAAGAACAAAGTTCTCACGATGTCGTGGTTCAGAACGGGACAAACTTCTCTGAGAG
ENSORLG00000015952	0.85	per3	-0.34	tds	tumor	CAAATCAACTGCGTGGACAACATCTTTAGATATTTGGAGGGCTGTACAACTTCAGTCCTAAAGCGGAAGAGTGACTCTCATTCCTTGGCCACCTCCTCTT
ENSORLG00000016829	0.71	tnfrsf14	-0.27	tds	tumor	AAAGTCAGAAACCTGGACATCTTTGTTTCTCCTGATGATGCTGATGAGAGTCTCCAGAGTTCTCTCTTGTCATCAGACAGAGTATGAGATCGGCGGTGAA
ENSORLG00000016946	0.81	slc38a11	0.45	tds	tumor	CTTTCATTGCCATGATAAGCTACAACATTACAACAGGTGACACCCTCACCAAGGTGTTTCAGAGAATCCCAGGAGTTGGTCCAGATCACATACTGGCAGA
ENSORLG00000017109	2.00	uncharacteirzed.24	1.63	tds	tumor	GCGGCGGAAACCTGTTTCATCCAAAAGAACTCCAACAATGGCTCAAAGTCCAACTGTAGACCAATTGTGCATGCCATCTACTAGTGGACAGCAGAGTGTG
ENSORLG00000017153	2.00	orla-uha	-4.86	tds	tumor	GGAGTAAAGAAAGGACAGATCCTCCCCAACAATGATGGAACTTTCCAGATGAGCGTTGATCTCCAACTTCCACCAGAAGCAGAAACGCATAGATATGAAT
ENSORLG00000017411	0.95	nots	-1.56	tds	tumor	CATCTCCTTGGAGTTATGGGCAAAGACACTCTAAGGATCGGTAATCTGACCGTCCTCAACCAAGAGTTTGGAGAGTCTGTGTATGAACCCGGCTCCACCT
ENSORLG00000017942	0.78	apoda.2	-2.12	tds	tumor	TGGTTTGAGATCCAGAGGCTGCCAACGAGCTTCCAGGTGGGTCAGTGCAGCACGGCCTTCTACACCCCGAAGGCTCCTGGAGTCATCGAGGTCCTGAACT
ENSORLG00000018272	2.00	flnb	0.67	tds	tumor	CATCGACTCCAAAGCCATTGTGGATGGAAACCTGAAGTTGATTCTGGGTTTGATCTGGACCCTCATTTTGCATTACTCCATCTCTATGCCTATGTGGGAG
ENSORLG00000018315	1.00	uncharacterized.2	-1.79	tds	tumor	CCAAACATGCTGGAAAACATTCGTACCATTGCAGACCAATGTGTGATACTAGCAGACAGTGTTGAAAAGCGGTATGCTGATGTCATTAATTTGGTCCAGG
ENSORLG00000018856	2.00	h2-ab	-1.38	tds	tumor	CTTGTCTCTGGCTGGATTCATCTACTACAAGAGGAAGGCCAGAGGTCGTATTCTGGTTCCCAGCAGCTGAGGTTCTTCTGTTCCTGCAGACAGATGGGTC
ENSORLG00000018864	2.00	mpz	-6.34	tds	tumor	AAGAGTAGGAGACAAAGAAGGAAAGAATGAGGATGGAGGAAGAGATGAAGAAGGACTGAGTACAGGAGGGATCATTGGGACTGCTCTGGCAGTTCTTGCT
ENSORLG00000019168	0.71	olfm4	0.70	tds	tumor	TGCAGACCTGCGAGGCCAGTGTGATCAACCTGCTGTCACAGGTGATGGTCTCCAACGAGAGTCTTCCTGACATCCAGGCCCAGATGGATAACGCGACGGC
ENSORLG00000019328	2.00	frrs1	-7.04	tds	tumor	TGCAAAAGAGGAAGAATTCAGTGTGTGGAGAATAATTTACAGTCTTCACATTTCTCTTTTATTCAGTTGCCAACAGCAAACCTGAACAGAGCAAAGCAAC
ENSORLG00000019336	0.85	frrs1.2	0.86	tds	tumor	CAGCTGTCTCCCAAACTTCCGCCTCTACAAAGACTGAAATCCAAGTGACGTGGCAACCCAGCCCATCCCAAGTGAAAGACATTGAGTTCCACGCAACTTT
ENSORLG00000019355	0.97	lei.1	-2.20	tds	tumor	TCTGGTGTTGGTCAACGCCATCTACTTCAAGGGAACCTGGGATCAACAGTTTCTGGGTATTCGGACGGAAGATGCTGAGTTTAGACTCAATAAGAAAGAC
ENSORLG00000019589	0.84	il4i1.2	-4.57	tds	tumor	GCCATGAGGATCCCATCAACTCACCAGATCCTTCTCTCACTTGTGAAGAAGCTGAATCTCACAACCAACAAATTCATCATGGACGATAAGGAGACCTTCT
ENSORLG00000019686	0.85	uncharacterized	-0.81	tds	tumor	CGGGAAAGGCGACCCAGAATGGCAGAAAACAGAGAAGACCCCTCCAGAGACCTCATAAACCTTCGCACAGAACACCCCTCTGCACCACTGGTAAGTGAAG
ENSORLG00000019793	2.00	mog	-0.81	tds	tumor	GGTCAAGCCCGATCCGGCGGATCCACAGGGCCGCGTCCCTTTTGTGTATTTTTACCGGAACGGGCAGACCATGACCACAGTTATGATGGAGTCGTTGATC
ENSORLG00000019887	2.00	mog.2	-1.33	tds	tumor	GTCGCTCCAGACCGGCTCTCTGGATCCTTCTTGGACTTTTTCCCTCAGTGATCCTCTCTGCTGTTTGTGTTTGTGTCTGTCAGAGGAAACAACCAGAAGG
ENSORLG00000019909	2.00	flncb	0.78	tds	tumor	GGATGGTACTCACACTGTCACCTACACTCCTGCTAACGATGTCTTGTACACCATTAATGTGAAGTACGCTGACCAGCAAGTGCCGCGCAGTCCTTTCAAA

ENSORLG00000019947	0.71	cyp2j6	-1.84	tds	tumor	AAGGAGTCTTATGGAAACGTCTACAGTTTTTTCATCGGTTCAAGACCAGCTGTTGTTGTAAATGGCCTACAAGCCATGAAGGAGGCAATCATGATCAAAG
ENSORLG00000020013	0.73	PFK-2/FBPase-2	-0.59	tds	tumor	AGCCATATTTTCCGCTTCTGTGTGACCTGCACTGCACACTCTATTATGACAGAGAGGATGATCAACTGTACCAGAAAGAATTTTATCAGGTAGAAGGCCA
ENSORLG00000020643	0.81	ddx17.2	0.47	tds	tumor	GGACAAGCTGAGTTCATGTTTGTGGACCTGACGTCTCTGAAGTCGGTGAGACATTTCGCGCAGGCCTTCAGGGACACGGGTCTACCTCTCCACGTGCTGG
ENSORLG00000020751	0.81	zfp184	0.15	tds	tumor	TCCCTGTTGCTGTTTAATCCACTGACTCTTGACATGGGGAGTAAAATGTCCTCCAGCGGAGGAAGCAGTGAGCAAAATACAAACTCTGACTTAACTTCTG
Xmrc	2.00	xmrk	7.53	tds	tumor	GGAATCGGGTCTCTGAGCAACACTATTGCTGTCAACTCAACCAACATCAGGTCCTTCAGCAACTGCACCAAGATCAACGGCGACATCATCCTCAACAGGA
ENSORLG00000000312	0	arhgap32b	0.24	housekeeper	housekeeper	GGGCGCCCAACCTACTGAGGTCCAAACAGATCGAGTCTGCCTGCTTCAGTGGCACGGCCGCCTTCATGGAGGTCCGGATCCAGTCTGTGGTGGTGGAGTT
ENSORLG00000003752	0	aif1l	-0.26	housekeeper	housekeeper	TGCCAGAGAAACTAGAGGGCTTCAAAAACAAATATGCTGAGTTCGACCTGAACGACGAGGGAGAGATCGAACTGATGGGTCTGAAGCGGATGATGGAGAA
ENSORLG00000003824	0	tdrd7	0.25	housekeeper	housekeeper	AGCAGAGTGTTGGACATCCTGTTCGTGGATGTTGGCGTTCAGGCGTCTGTGGAGGTTTTTGAGCTGAGAGAGATCCCGCCGATGTTCCTCCGTGACTTTG
ENSORLG00000004424	0	asph	-0.22	housekeeper	housekeeper	GTGACAAAGTCCGCTCCAGTCCGAGTCCAGGTCCCGCGGTCCAGCATGGCTCCGAAGAAAGCGGCCAAGTCTAGCCACAAGTCTAAAGATGGCCCTAAAA
ENSORLG00000005412	0	amot	-0.22	housekeeper	housekeeper	AACCGCAAGACCATCTCACAGCTGCTCACACAGAATAAAGAAATGCAGCGTGAGAAAGAGAAGCTGGAAATGGAGTTAAACGCCCTTCGCTCCACTGCAG
ENSORLG00000006320	0	caspa	-0.24	housekeeper	housekeeper	CAGACAGATGGCGACCATCGAGAGATGCACGCTGCTAAAGCAATTCTACTTCTATCCAGGCCTTGGAGACACAAACCATTCATGATGCAAAGTTTACAAC
ENSORLG00000007168	0	efemp1	0.26	housekeeper	housekeeper	TTTCGGACCATTCTCTGTAAACACATAGATGAATGTGCAACAGGCCGGCACACTTGTGGACCTGAACAGACATGCTACAACACCAGAGGGTCTTTCATGT
ENSORLG00000007781	0	c20orf27	-0.25	housekeeper	housekeeper	TGTTCATTTTGATGAGAAACTGCATGACTCGGTGGTCATGGTGACTCAGGAGGATGATGGCAACTTCATGGTGAAGGTTGGCTTCTTGAAGACCCAGCAT
ENSORLG00000007872	0	mpp6a	0.25	housekeeper	housekeeper	TCTGAAGCCTTTCTTCAACTATAATCCAGCCACGGACAACCTAATTCCCTGCAAAGAGGCGGGCCTTCCCTTTTCTAAAGGAGAAATTCTTCATGTGGTC
ENSORLG00000008280	0	capn1	0.24	housekeeper	housekeeper	TAGCTCTGTCAGGGATCGACTACAAAACCGCAGTGAAGACGGTGAATTTTGGATGGCATTCAGTGACTTTCTGCGGGAGTTCAGTCGTGTGGAAATCTGC
ENSORLG00000009316	0	serpine2	0.24	housekeeper	housekeeper	GAACACCAAGATGCGACCCTTCCACACGGCTGACGGAAGCGTTTCTAAAGTTCCAATGATGTCCCAGCTGTCGCTCTTTAACATCGGCATGGCCACCACC
ENSORLG00000010791	0	zfpl1	0.25	housekeeper	housekeeper	GTGGTCACCTTTTCTACTGCCAACACGTGCGACACTGCTACAATCCACACAGCGTCTTCACCAAGAAAAATCTACGACACGCGGGACGTAGCTCACAGCT
ENSORLG00000011874	0	rbm25b	0.25	housekeeper	housekeeper	TTCCGGTTCATATGGGCATCATGGCACCTGCTTCTGCAGTAATGATGTCATCCGTTCCAGTTGTCAGTAAACCTCCGGTTCCAAAGAGGGATCTTTCTGC
ENSORLG00000012542	0	jupa	0.24	housekeeper	housekeeper	GCCTGTCCGACATCCACAGCAGGCCCGTCAACCAGACTCTGACATTTTTTTATGACTTAGTTGCAAGAGGCCGAAATGTGATGAAGGCTTTTAGAATCCC
ENSORLG00000012754	0	tmem55a	0.26	housekeeper	housekeeper	CCCAGGAGGCGCTGTTGCGCTTACATTACTGTAGGAATGATCTGCATTTTCATTGGTGTGGGTTTAACAGTCGGGACTCGAGGTTTTGCCCGCCGTTACA
ENSORLG00000012984	0	wdr45b	0.23	housekeeper	housekeeper	AGAACAAACTTTACTCTGTGGGTCTCCCAAATTTCTATGAGAAAGAGGGCAAAGTCAAATGTGAGAAGAAAGTGCCCTATTGCAGAGTGGGAGCAAACAC
ENSORLG00000013918	0	ggh	-0.24	housekeeper	housekeeper	TCTATCCTCACTCTCCGTCTGCAGTCAAGACCTCCTTCTACATGGCGGAGTTCTTCATCGGTGAAGCCAGGAAGAGCTTCCACAGATTTGAATCAAAGGA
ENSORLG00000014242	0	fam45a	-0.25	housekeeper	housekeeper	GCAGGATTTGTAGATCCAGAAGTGAGCAACAGACCGGACTTGTTTGATGTTTTCGTGAGTCTTCCAGACGGTGTCATCACCGTGTCCCAGAGTGCTAAAG
ENSORLG00000014497	0	ddx17	0.22	housekeeper	housekeeper	CTCAGACTGGCTCTGGGAAGACTTTAGCGTATCTCCTGCCTGCCATAGTTCACATCAATCATCAGCCTTACTTGGATCGAGGAGATGGACCCATTTGTCT
ENSORLG00000015042	0	prph	-0.24	housekeeper	housekeeper	CCCATCTATAATATTGGGATGAGCAATCACCTTGGTGAACGAGATTACGAAAGAATTCCAGACTCCCAGAGCAAAAGAACCGTGGTGATAAAGACTGTGG
ENSORLG00000015424	0	cab39	0.24	housekeeper	housekeeper	AAAATCACGCTGGGGTCAGAGCAGTTCTATGACTTCTTCAGATACGTGGAGATGTCGACGTTTGACATCGCTTCAGACGCTTTTGCCACCTTCAAGGACC
ENSORLG00000015716	0	ctsk	-0.24	housekeeper	housekeeper	GGGCATGGTGACTTCTGTGAAGAACCAGGGTTCATGTGGTTCCTGCTGGGCTTTCAGCTCTGTAGGAGCCCTGGAGGGCCAGTTGGCTAAAAAGACTGGC
ENSORLG00000015837	0	gap43	-0.22	housekeeper	housekeeper	TAGCTTCCGTGGACACATAACTCGGAAAAAGATGAAAGACGGAGAGGAGGAGAAGGAAGAAAACAGTCCTGTTGCAGATGAGGCAAAAGAGAGAGAAGAG
ENSORLG00000016109	0	fsd1l	-0.25	housekeeper	housekeeper	TGATATCACAGAGGCGCAGGTCACCATCTCAGGTCTAAAGTTCGACTCACAGTTTGTCGTCGTTCGAGTTCGAGCCAGAAACAAAGCAGCAGCTGGAGAA
ENSORLG00000018359	0	szrd1	0.24	housekeeper	housekeeper	CGGGGAAATTGAAAGAAGACTTGAGGCAAAGCTAAAGATAAATCAGGAGGCAAAGAAGTCCACTTTGAAATCTGCGGGTTCACCTGTGCGAACCGCCGTT
ENSORLG00000019528	0	fbxo28	0.23	housekeeper	housekeeper	ATCCTCAACTTCCTGTCCTATGACGAGATTAGCCTTCTGCGTGCGGTGTGCAAGCGTATGGACACGATCTGCCAGCGTGTCCTGAACCAAGGCTTTTTGA
ENSORLG00000019941	0	spns1	-0.23	housekeeper	housekeeper	CTCTGCTCACCGTCTTGGTCCTCTGCTACATCAACCTGCTGAACTACATGGACCGCTTTACCGTGGCGGGCGTCCTCCCAGAGATCGAGAATTTCTTCGG
ENSORLG00000020136	0	epha2	-0.24	housekeeper	housekeeper	CTCGGACGGTTCTCCCTTCAGGTCCGTGTCCGAATGGCTGGAGTCCATTAAAATGAACCAGTACATTGACAACTTCACATCTGCTGGGATACTCACCATG
ENSORLG00000021779	0	nd5	-0.25	housekeeper	housekeeper	ACTGGCTTTCCTACACATCTGCACCCATGCCTTCTTCAAAGCAATACTTTTCTTATGTTCCGGCTCCATCATTCACAGCCTTAACGACGAGCAGGACATC
ENSORLG00000000317	0	cyclin d1	-0.18	reference	cell cycle	TGGATGTTAGAGGTCTGTGAGGAACAGAAATGCGAGGAGGAGGTTTTTCCGTTGGCAATGAACTATTTGGACAGATTTTTATCAGTGGAGCCCACCAGGA
ENSORLG00000001112	0	wee1	0.01	reference	cell cycle	AAAATAGGTGATCTTGGTCATGTGACTACAGCGAACAATCCACAGGTGGAGGAAGGTGACAGTCGATATTTGGCTAATGAAGTTTTGCAAGAGGACTACA
ENSORLG00000007021	0	mycb	0.52	reference	cell cycle	GACGCCACGGAGGTGTGCCCTCACTCCTCTGGTCCTCAAACGCTCTCACATCAACATCCACCAACACAACTATGCCGCCAAACAGCCTGCTGCGAAGCGG
ENSORLG00000010987	0	cenpf	-0.49	reference	cell cycle	GAAGCTCCCATCAAATGGAACAGCTGAAAACTCTGAATCAAGGTGTTCTTTCGTTTTCCTCAGAACTTCATGGCCGTGTGTCAGAACTGGAGAGGAATCT
ENSORLG00000011120	0	numa1	-0.49	reference	cell cycle	TGTTAATAGTTCTCTTAAGGCTGCTGAGGAGCAAGTCAAAGCCAAGGAAGGTCTGATGGCCAAGCAGGATGAGGAGAACTCTTTACAGATTGAAGAACAT
ENSORLG00000013657	0	cyclin b1	0.48	reference	cell cycle	TGCAGGGGAATGGGATGTGACACTGCAGCATTACATGGAGTACACAGCAGAGACCCTGACACCTGTTATGGCGCACATCGCTAAGAATGTCGTGAAAGTC
ENSORLG00000017163	0	mdm2	0.29	reference	cell cycle	CTCGAAACGCGCACACCCCACTTCCTGTTTCCGCCCATAACCCGGACTTTGTGTTCAGTCGGAAAGAGTCGGACAGCGACAACTTCAGCGTGGAGTTCGA
ENSORLG00000004495	0	clockb	0.12	reference	circadian	CTTGGAGATGCTAGCTAAATGCCATGAACACTTATTGCAGTATGGGAAAGGAAAGTCATGCTACTACCGTTTTCTAACAAAAGGTCAACAGTGGATCTGG
ENSORLG00000005149	0	aanat2	-0.13	reference	circadian	AGCCTTGTGTGTACTGTGGTGTTAGTATCATGACTCAGAAGGTCAGCGGCTCGCCTTTCCTCAAGCCTTTCTTTCTGAAGACGCCCGTCAGAGTTGCGAC
ENSORLG00000005458	0	raver1	-0.50	reference	circadian	CATCTGAACAGAGCTGTTGGAGCGCCTCCTGTGAGCCACAATGACTATCACCCAGGTCTGGGCAATCCTGCAAAGACTCCCATTGGAAGCCACAAGCGCG
ENSORLG00000005554	0	agt	0.00	reference	circadian	GCTCTTTATTTAGTTTCTACCTGGGAGCTTCCAAGACGACGGCAAGCTATTTTCTGCGTCTTCTGGGCCTCAGTGACACCGACAGAGAGGAATGTTTGTC
ENSORLG00000006929	0	per1	-0.38	reference	circadian	CCAAGGCAGAGAGTGTAGCCTCAGTTACGTCTCAGTGTAGCTTCAGCAGCACCATCGTCCATGTAGGAGACAAGAAACCTCCCGAGTCAGATATAGTCAT
ENSORLG00000007645	0	ror	0.12	reference	circadian	GGTGGAGTTTGCCAAGCGCATCGATGGCTTCATGGAGCTGTGTCAGAACGATCAGATAGTGCTGCTGAAAGCAGGCTCTCTGGAAGTTGTGTTTGTCAGA

ENSORLG00000008302	0	dlgap1a	-0.08	reference	circadian	CCCAGAACCTAACAGGCACATCCTGTCACCTAACGCTCTGTCTCGACAATATTCCCGTGATGCTGCAACGTCCACAGTCAGCATTCAGGGCTCAGGGAAC
ENSORLG00000009426	0	npas2	0.28	reference	circadian	AGGCTCAGGAGCTGTACTTGGACATCTGCTCGACTCTGGATGCTTCGCGGGACAGAAACAGCGGTGCACGTTCAGTGTCCTCCCAGAGCTCCCGGAAGTC
ENSORLG00000010388	0	dlgap1b	-0.12	reference	circadian	AGAGGTGTACCAGAAGGCATCAGTCAACCTCGATAAAGCTCTTGTGAAAGCTGAGCCAGGACGCACGTGCCATTTCCTTCAGGTACCTCAGGATGATTGG
ENSORLG00000015066	0	timeless	-0.06	reference	circadian	CTCACATGACGAAGGCATCCGGCAGAGTTCCAATGTTCTAAAAAGTAACATTTTCTACATGATGGAGTTCAGGGAGATCTTCCTGACCTTGCTGAGAAAA
ENSORLG00000015456	0	per2	-0.23	reference	circadian	GCACAAAGTGCGCATGGGACCCGTCAACGAAGATGTTTTTGCAGCTCCGGCTTTCCACGAAGGGAAGATGATGGATTCCGACATCCAAGAAATAAGCGAG
ENSORLG00000016382	0	cry1ab	-0.45	reference	circadian	AAGGTCTTTGAGGAGCTGTTGTTGGATGCAGACTGGAGTGTCAATGCAGGCAGCTGGATGTGGCTCTCCTGCAGCTCCTTCTTCCAGCAGTTCTTCCACT
ENSORLG00000016410	0	cry1aa	-0.13	reference	circadian	CAACTCTCGTCTCTTTGTGATCCGAGGCCAACCCACCGATGTCTTTCCCAGACTTTTCAAGGAATGGAAGATCGCTCGCCTGTCTTACGAGTACGACTCT
ENSORLG00000016431	0	nr1d2a	0.08	reference	circadian	CTGCACCATCATGAGGATAAACCGCAACCGCTGCCAGCAGTGTCGCTTCAAGAAGTGCCTGGCCGTGGGCATGTCCAGAGACGCTGTGAGGTTCGGCCGC
ENSORLG00000016612	0	per2.2	0.02	reference	circadian	CCACACAGCAAGGCTTTATGGGGGCCCAAAATTCTCCTTTTGCTCCCATGATGGCACCTGTTGTTGCTTTTCTTATGCCTAACTACCTGTACCCTGCAAT
ENSORLG00000019097	0	cry1ba	0.29	reference	circadian	GCCTCTCCTGTCGCCTTTTCTACTTCAAGCTCACTGACCTCTACCGCAAGGTAAAAAAGAACAGCTCTCCTCCACTCTCCCTGTACGGCCAGTTACTGTG
ENSORLG00000019370	0	arntl1a	0.35	reference	circadian	TTTAACACTGACGATATGATAAACATCTGTGATGACCTGATGGCCGACCAGCGGATGGACATCTCGTCTACCATTACTGACTTCATGTCTCCAGGCTCCA
ENSORLG00000001780	0	sox3	-0.03	reference	development	TCTCACCGACGCAGAGAAGAGGCCGTTCATCGACGAGGCCAAGCGTCTGCGGGCGATGCACATGAAAGAACACCCGGATTACAAATACCGTCCACGCAGG
ENSORLG00000006269	0	ybx2	0.35	reference	development	ATCCAGCAGGTGTGCATTTGATGTTGCATAATGGGAGCCAGTTTAGTTGTTACCTCCTAAAATTGTTGAAATGGTCAGTTCTCATCTCTGCTCAGCTGGA
ENSORLG00000003982	0	gadd45ga	0.20	reference	DNA repair	ATCGCATGATGATGTTAATGATGAGAAAGTCAAGCAGCCACGCTTCGACTGGGGGAAGTCGAGCCTTTCTGTTTCTTGATTAGGAGGCTGTCTGCACCTT
ENSORLG00000005244	0	gadd45a	0.16	reference	DNA repair	AGGAAGTTCTGACCTCCGCGCTGCCTCAGGGCGCCATCACTGTCGGTGTGTACGAGGCTGCCAAATCTCTCAACGTAGACCCGGATAACGTGGTTCTGTG
ENSORLG00000006390	0	tp53	-0.22	reference	DNA repair	AGGAGGAGGACAACAGAGAAGTCTTTCATTTTGAGGTTTATGGGAGGGAGCGCTACGAGTTCCTTAAGAAGATAAACGATGGCCTTGAACTGCTGGAAAA
ENSORLG00000013231	0	gadd45ba	-0.08	reference	DNA repair	ACTGACTTCAGCATGACTCTTGAGGAGGTTGTTGGATCCAGCAGTACCGACAAAAAGATGGAGACGGTGAGTCAGGCTCTGGAGGAGTTGCTGGTCGCGG
ENSORLG00000014316	0	gadd45bb	0.99	reference	DNA repair	TTCTTGGAGCTGCAGATGTCAATAAAGACCAAGACGAGAGAAAAGACCTCCATTGCTTGCTGGTCACGAACCCTCAGGCGAACCACTGCAGGCTGCAGGA
ENSORLG00000014766	0	gadd45g	-0.07	reference	DNA repair	CAATCCTGGAAAGTATTATAACTTCAATACTGCAGAGCTGCACGGCAACGACCTCACCATGACCGAGTGTGAAGTTTACCAAGTGGAAGAGACTGTTGAA
ENSORLG00000016457	0	parpbp	-0.60	reference	DNA repair	TTGGTTCGTCTGTGCAAAACAGAGACAATGAGATTACAGGTGAAAATGGCGGTGAGACGGGTTTTCTGCTCCTACCTGAGTTTGCTTATCAACTCCAAGA
ENSORLG00000000813	0	fancd2	-0.08	reference	Fanconi anemia	GTATGCCGTTTTTGTCAAGGGAATCTTGGACTACATGGACAACCTGACGTCTCAGCAGATTCGAAAACTTTTCCAGCTTCTAAGCAGACTGGCGTTCGGG
ENSORLG00000001695	0	fancl	-0.14	reference	Fanconi anemia	GACTCGCTGTTTAAAGACAACCCGCTTCTTTTGCCTGTGAACGAGCAGAAAACGGTTTATGATGGATTCATCTCTGTGCAGGACAAAGATCTACGAATCC
ENSORLG00000014821	0	fancj_brip1)	0.12	reference	Fanconi anemia	CCAGGTCTTGTTGTCTGAGCGTGGATATGAAAGTGCCAGTAAAGTCTGGAGCGGGAAAGACATACTGGGCATCTTTTATGGTTTGGGCATTACAGCTGAC
ENSORLG00000017835	0	fancb	0.05	reference	Fanconi anemia	TTATTTTGAGAATGGAATTGCAAAGGATGCCTGCCCACTGCCCTTTGAGCAAGCAGAAGACATTCAAGTGGCTGACTTGGGAAGAAACGGCACCCTTTTT
ENSORLG00000004345	0	ptch1	0.01	reference	hedgehog signaling	TCACCACCACCACTCTGGAAGATATCCTCAAGTCTTTTTCTGAAATCAGTGTTATCCGTGTGGCCAGTGGATACCTGCTGATGCTTGCTTATGCGTGTCT
ENSORLG00000005455	0	gli1	0.03	reference	hedgehog signaling	CGCTCCATGATGATTAGTCCTGGGGAGAGAAACTTCATGGGTTACCAGCAACAGAATCAAACCATTGGAAGTGGAGGTCCTATTTCAAATCAGCTATCCC
ENSORLG00000008275	0	gli2b	-0.22	reference	hedgehog signaling	TTTATTGGCAAGACAAACGACAAGCGAGCTTCCCCGGACCTTTCGGCGGATGAGCAACTGGCCTGCCGCTGGAAAAAATGTCACCTGCTTTTTGACTCCT
ENSORLG00000010364	0	gli2a	-0.21	reference	hedgehog signaling	GTACAAAATGTTGATTCACATCCGCACACACACCAATGAGAAGCCGCACCATTGCCCCACCTGTAACAAGAGCTTCTCACGTCTGGAGAACCTGAAGATT
ENSORLG00000016137	0	ptch2	0.01	reference	hedgehog signaling	TTTAACTACAGCCAGTTGACTTCTCGTCAGCTGTTGGACGCCGAGGGTTTGATCCCGCCTGAGGTGTTTTACATTTACCTGACTGTATGGGTCAGTAACG
ENSORLG00000004325	0	ucp3	-0.22	reference	light responsive	CCTACGGGGGCTTTGGAAAGGCGCTCTACCTAACATCATTCGTAACGCCACCGTAAACTGCTCTGAGCTGGTTACCTATGATGTCATCAAGGAACTCCTC
ENSORLG00000005665	0	klh38b	0.46	reference	light responsive	GGGTCTCGGACCAGACAGACGATTAACAAGCAGTGTTCAAAGGTACAACAGTATGTTCAATCAATGGGAGTGGATAGCGCCCATGCCGGAAGCCGTCCTA
ENSORLG00000017552	0	c5	0.00	reference	light responsive	TCTGGTGGCCTTATCAGCAGTGGACTCCGCCGTTTACAGTATACGCCGAAACTACAAAGACCCAATTTCCATGGTTCTGCGTCACATTGAACAAAGTGAC
ENSORLG00000007554	0	map2k6	-0.05	reference	MAPK signaling	ATACGGAGTTGTGGACAAGATGAAACACGTACCCACCAACGTTATCATGGCCGTCAAGAGGATTCGTGCCACCGTGAATACTTTGGAGCAGAAGAGGCTG
ENSORLG00000013838	0	map2k6.2	-0.10	reference	MAPK singaling	AGAAGAAACCAGTGCTCAAGCTCTCCAAGGAAGTGTTTGAGCAACCTGCACCTGCCGCAGTGCCGCCCAGAGATCTGGACTCAAAGGCTTGCGTAACAAT
ENSORLG00000001386	0	pomca	0.18	reference	pigmentation	AGATCCCTTCTCTTTCATCTCTCCATCCCCTCAAACCAAACGCTCCTACTCCATGGAGCACTTCCGATGGGGCAAGCCCGTCGGGCGCAAACGTCGCCCC
ENSORLG00000014880	0	pomcb	0.35	reference	pigmentation	GAGTCAGATATGAACCGTCGCTCATATGCCATGGAGCATTTCCGCTGGGGGAAACCCATAGGACGCAAACGGAGACCCATCAAGGTTTTTGCCTCCTCTC
ENSORLG00000002045	0	fosl1a	0.00	reference	proliferation	TTTCCCAAGACGAGCTGGAAAGGCGCAGAATAAGAAGAGAAAGGAATAAAATGGCTGCGGCGAAATGTCGCAATCGTCGCCGGGAGCTGACAGACTCGCT
ENSORLG00000010470	0	tnfb	0.00	reference	proliferation	CAGCGGTACTCTTCTTCAGCTCGCATACCAAGCATCCAGAAGTACACGAAGACTCATTTCATCTCAAACTCAGGCAGATTTCCAACGTGAGAGCAGCCAT
ENSORLG00000013777	0	kpna2	-0.39	reference	proliferation	AATGTGCTTTGGAGTCTCTGCGCAGGAGAGCGGAAGTGAAGGTGGAACTGAGAAAAGCAAAGAAAGATGAGCAGATTTTTAAGAGAAGAGCCATCCAACA
ENSORLG00000015192	0	Kpna2.2	-0.12	reference	proliferation	CAAAACCGTCCTCGTCATCCTGGATGCAATAACCAACATCTTTTTGGCTGGAGATAAGATCGGGGAGTCCGACAAACTCAGCCTAATGATTGAAGAATTT
ENSORLG00000017504	0	fosaa	0.00	reference	proliferation	TGTTCTCCTTTAATTACTCTGAGATTGAATCCTTGGCTGGCGGCTGCGACAGCGTCAAAGGCGGCCTGGCTGCGTCTGAATTAATCAAAGATAGTCTCAA
ENSORLG00000000284	0	cdkn1a	0.39	reference	toxicity	TGGAGTACCAGGCCGCCCTGCGGAAAGACCTGGACGAAGCGTCCCTTCGCTGGGGCTTCGACTTCCGCTCCGACAAGCCCCTGGAGGGCAGCCACTTCCT
ENSORLG00000000624	0	gsr	-0.03	reference	toxicity	TTCTCAGGTTAACGTTGGCTGTGTTCCTAAGAAGGTTATGTGGAATGCAGCTGTTCATGCAGAGTATCTACACGACCACTGTGACTACGGCTTTGAGACT
ENSORLG00000000823	0	gpx1b	-0.16	reference	toxicity	CCCGGGAATGGCTTCGAGCCAAAGTTCCAGCTCTTGGAGAAGGTGGATGTGAACGGGAAGGATGCCCACCCTTTGTTTGCCTTTCTGAGGGAGCAGCTCC
ENSORLG00000000945	0	e2f1	-0.26	reference	toxicity	ACCTCCTTCTACTCCTGCTCCTCCCAGAGTTCCTCGAAGCACGGCGGAGAAGTCGAGGTACGACACCTCCCTGAACCTGACCACCAAGCGCTTCCTCAAC
ENSORLG00000001050	0	nos2	0.46	reference	toxicity	ATGTCAGGTAGCATAACGCCAGTTTTTCACCAAGAAATGCTCAACTACCGCCTCACTCCTTCCTTTGAGTATCAGGCTGATCCGTGGAACACTTACGTAT
ENSORLG00000001399	0	ddb1	-0.02	reference	toxicity	GTCTTTGCCTGCTCTGACCGACCCACGGTCATCTACTCATCCAATCACAAACTGGTGTTTTCCAATGTCAACCTCAAGGAGGTCAATTACATGTGCCCAC

ENSORLG00000001624	0	ephx2	0.04	reference	toxicity	CCCGTTCTGCAGCCCAGACGAAGTTTGTCACGGATACGTCACCATCAGAACTGGTGTGAGGTCTCACTTTGTGGAGATGGGCTGCGGTCCTCCTGTTCTG
ENSORLG00000001678	0	rad50	-0.01	reference	toxicity	GGCAACATGCAGGTCCTACAACTGCGCCAAGAACGTCGTGACGTGGAACGCAAATTGGAGGATTTAAAGAAGAACCGCAGTATTGCACTGGGCCGCCAGA
ENSORLG00000001746	0	cat	0.03	reference	toxicity	CGGCTACCGCCACATGAACGGCTACGGCTCCCACACTTTCAAGCTGGTCAACGCTGACGGCGACCGCGTCTACTGCAAGTTCCATTACAAGACTGACCAA
ENSORLG00000002937	0	pcna	-0.14	reference	toxicity	ATGCCCTCCGGGGAGTTCGCCCGTATTTGCCGTGACCTGTCGCAGATCGGCGACGCCGTCATGATCTCCTGTGCCAAAGACGGTGTTAAATTCTCCGCTT
ENSORLG00000003753	0	hmox1	0.04	reference	toxicity	TTCAACATCCAGGTCTTTGATGACATTGAGAAGCTGCTGGACGTCACAGAAAACAAGCTGCAGAAGATTTTAACCTTCAAGTCAGGAGAGGCGAAAACAC
ENSORLG00000003850	0	mif	0.03	reference	toxicity	GGCATCTGTCAAGACAGGATCTATGTTAACTTTGTTGACATGGATGCAGCCAATGTGGGCTGGAACAACACTACATTTGGCTAAGATGGAAGATACTGTC
ENSORLG00000004389	0	sod1	-0.07	reference	toxicity	GACCGCAGGAGATAATAACGTTGCTAAAATAGATATCACGGACAAACTTATCAGACTCTCAGGTCCAGACTCTATTGTTGGCAGAACTGTGGTGGTCCAT
ENSORLG00000004393	0	ercc3	-0.05	reference	toxicity	TCGTCGGGTTCTCACCATCGTCCAAGCTCATTGCAAACTGGGCCTCACTGCCACACTGGTCAGAGAAGATGACAAGATTGTGGATCTGAACTTCTTGATA
ENSORLG00000004568	0	baxa	0.10	reference	toxicity	ACCAGACATCATTAGAACTATATTCACCTGGACCATAGATTACCTTCGGGATTATGTGATTGCCTGGATAAGAGAGCAAGGCGGCTGGGATGGCATCTTT
ENSORLG00000005095	0	ptgs1	0.13	reference	toxicity	GCCGTGAGTACAATCAAGGAGTCCCGCCAGCTCCGGATCCAGCCTTTCAACGAGTACAGGAAGCGTTTTAACCTGGAGCCGTACGCGTCTTTCAGAGAGT
ENSORLG00000005361	0	ddit3	0.12	reference	toxicity	GAGTTCTTGGATGTTCTGGAAAGTTGTTCTCTCACCTGGCTGACTGATGGAAATCAGTCGATGAGTGAAAGCGTCCACAGAACAACAGAGGAGATCCATA
ENSORLG00000005451	0	egr1	-0.03	reference	toxicity	CAATCAACCCTCCCTCACTCCTCTGTCCACCATCAAAGCATTTGCCACCCAGACTGGTTCCCAGGACTTAAAAGGTGTCTATCAGTCGCAGCTAATTAAA
ENSORLG00000005891	0	cryab	0.73	reference	toxicity	GTTCCTGAGAAAGTACAGGCTCCCATTCGGTGTGACAGGAGCCAACGTTACCTCGAACCTCACCTTCGATGGCGTGCTGACCATCACGGCGCCTCGCTCC
ENSORLG00000005961	0	gstm1	-0.03	reference	toxicity	AGAACGTGGTCATGGACTTCAGAATGAGCTTTATCAGGATGTGCTACACTGACTACGAAAACCTGAAGCCAGAGTACCTCAAGACGCTGCCTGATGTTTT
ENSORLG00000006260	0	dnaja1	0.03	reference	toxicity	ACTCTGTTCGTCACATCACATCCAGGAGAATTAATAAAGCCTGAAGACACCAAATGTATCCTCAATGAAGGGATGCCCATTTACCGCCGACCCTTTGAGA
ENSORLG00000007126	0	ung	0.04	reference	toxicity	CAGGTCAAGCGCATGGATTGTGTTTCAGCGTGAAACGACCCATTTCTCCTCCACCCAGCTTGGAGAACATGTACAAAGAACTGGTTTCTGATATTGAAGG
ENSORLG00000007920	0	cyp7a1	0.03	reference	toxicity	CAGAACATGTCAGATCTCATCTCCATGAGGATGATTCTGAACGACTCCCTATCCACTTTTAACGATGTCAGTAAAGCCAGGACCCATGTGGCTCTGCTGT
ENSORLG00000010473	0	serpine1	0.33	reference	toxicity	TGCTGACTTCACACGGATAACCTCTGATGAGAGGCTCTGCGTGTCCAAAGTCCTGCAGAGAGTAACGATTGAGGTGAACGAACAAGGAACCAAGGCAGCA
ENSORLG00000010533	0	prdx1	0.10	reference	toxicity	CCTTTGTGTGTCCCACTGAGATCATCGCCTTCAGCGACGCTGCTGACGACTTCAGGAAGATTGGCTGTGAAGTCATCGCTGCCTCTGTGGACTCTCACTT
ENSORLG00000011198	0	casp8	-0.06	reference	toxicity	GACGTCTCTGGGCTTTATAAACTGCTCTTGGAGTTTGATAAAAAGCTGGCAGCAGTTGTCGAGAAGTACATGCAAGATCATCGTGGAGCATTAACTGATA
ENSORLG00000011398	0	porb	0.12	reference	toxicity	CTGAAGCTCCACAACGACGTCAACATGAACAAAGTGTTCACCGGAGAAATCGGCCGCCTGAAGAGTTTTGAGGTCCAAAAGCCGCCTTTTGATGCTAAAA
ENSORLG00000011849	0	tnfsf10	-0.65	reference	toxicity	GGTCACTCAACAGCTCCACTACCTCATAGAGAAGAAAATTGCTGACAGTTTCCAGAAGGAGATTTCCAGCGCTGTGTCAAATAAGCTAACAGGTGTTCTG
ENSORLG00000012828	0	atm	0.01	reference	toxicity	AGGCGCCATGTCTAGTCTCAGATCGGTTATGAAGCTGTGCACTCAGTCCGCAAGCAGGAAACCTGTGGTCCACGTTAATGCCGTCTCCTCTGGTTTGTTC
ENSORLG00000013261	0	sod2	-0.06	reference	toxicity	GACTGCACAGGTTACCCTTCAGCCTGCTCTGAAGTTTAATGGAGGAGGTCACATTAACCACACTATCTTCTGGACAAACCTTTCTCCCAATGGTGGAGGC
ENSORLG00000013974	0	bcl2l1	0.10	reference	toxicity	TCAAGGTTTTAGTGATCTCTCCGTGCAGCTTCATATCACTCCAGACACGGCCTACCAAAACTTCAAAAGGGTGTTGGATGAGCTGTTCAAGGATGGGATC
ENSORLG00000014421	0	cyp1a	0.25	reference	toxicity	GTGGCAGGATCCATCATCCTTTAACCCAGATCGTTTCCTGAGTGAAGATGGAAGTGAGGTTAATCGGCTAGACGGAGAGAAAGTGATGGTTTTTGGTCTG
ENSORLG00000014528	0	prdx2	-0.04	reference	toxicity	ACAGAGGTCTGTTTGTGATCGACGGCAACGGCGTCTTGAGACAGATCACCATTAACGATTTGCCCGTGGGTCGCTCCGTGGATGAAACTCTCCGTCTGAT
ENSORLG00000015186	0	xrcc1	-0.10	reference	toxicity	ACGCCCAGCCCAAGCACCACTAAAGTGTCGTCCACTCAGAGATCCACTGAGAAGAAGAAGGAGGTACAGTCTAAACCGGAGCTTAAACCCAAACCAAAAA
ENSORLG00000016225	0	ercc1	-0.29	reference	toxicity	GTAAATCATCCACCAAAGTTGGTGCAGCAGCCCCTCTCCTATGCAGAATACATAGTTCAGAGTAAAGGCAGTGTGGTTACTGTACCCAAGAACTTTCCCT
ENSORLG00000016606	0	rad23aa	-0.04	reference	toxicity	CTCTGGCTCCACATCAACCGCTGCCCCGCCTTCAAATCCTCCTGCTGCACCCACCCCTGCAGCTGTCCCTATTCCTCCTGAGGAGGCCAAACAGGAGCAA
ENSORLG00000016636	0	igfbp6b	0.34	reference	toxicity	CCACCCCACAGGTCCTCATCCCTCACACAGTGGCGACATTGAAAAAGCGCCCTGCCGCAAGCTGCTCAACAGCGTGTTGAGGGGCCTTGAGCTGACAATC
ENSORLG00000020086	0	anxa5a	-0.01	reference	toxicity	TGGGAATTTGGAGAATTTACTGCTAGCTGTTGTGAAATGTGCCAGGAGTGTTCCTGACTTTTTTGCTGAAACCCTTTACAAGTCCATGAGGAGAGCTGGA
ENSORLG00000020130	0	chek2	-0.07	reference	toxicity	GTCGGAGCAGATCATTCGAGGAGAGTTCACTATGGTTCCCTACAAGTGGAAACACATTTCAAACCAAGCAAAGGACATGGTGAGGAAGCTGCTGGTGGTC
ENSORLG00000020478	0	ugt1a1	0.05	reference	toxicity	TGACGATCTGGTTAGCAGGCGCATCGAAAGTGGCATGTCCTACAAAGATCTCCTGAGCCACGGGGCGATCTGGCTCTTCAGATATGATTTCACTTTTGAA
ENSORLG00000000217	0	il1b	0.31	reference	wavelength	CAGCACAGCTCACAGCAAGCACACAGCAACACAGGATCTGACAGGTCTTTCTTTTCTCTGAACACCACGGAATCTGAGATGGCGTGCCACAAGAGCGACA
ENSORLG00000000577	0	usp25	0.22	reference	wavelength	CATAAAGTTGGAATACGCGCGACTTGTGAGGTTTGCCCAAGAAGGCCCGACTCCTGAGAACGACTACCGACTACAGCACATTATCGTCTACTTCATCCAG
ENSORLG00000002966	0	f8	-0.51	reference	wavelength	AGAGTTCCACGCCGTGCACTTCCACGGTTTGCCTTTCACTGTTCACACCAAGCAGGAGCACCGGATGGGGGTGTTCAGCCTTTTTCCAGGTGTTTTTGGG
ENSORLG00000003181	0	noxo1b	-0.58	reference	wavelength	GCGGCTCTGGCCACCAAGACCCGGCTTCAGATCCAACCGGAGCCCATTTACAGCCGCTAAGCCAAGAAAATCCGATGAACTGGAGACTCTCCTCTATTTC
ENSORLG00000003433	0	XIRP2	-0.02	reference	wavelength	TAAGCAAATGTGAAAGGACGGAGAAAATCCAAGTTGAAGGCGTCACAAGTGGATCTGTAAAGTCCAACAAGGATCTTTTTGAATCCACACCGCTCTATGC
ENSORLG00000004247	0	finca	-0.02	reference	wavelength	GAATCTACGCACAGGATGTAGAAGGTTGTACCATCGACATCAAGATCACTGACAGGGGCGACGGAACATTCATGTGTGTGTATACTCCTGTTAAACCCAT
ENSORLG00000005687	0	usp13	-0.04	reference	wavelength	TCCACCATGTCTGGGCATTATGTCTGTCATATCAAAAAGGAGGGGAGGTGGGTGATCTACAATGACCACAAAGTGTGTTTGTCAGAAAGACCTCCAAAAG
ENSORLG00000006356	0	smox	0.11	reference	wavelength	TGTACAACGAGGTGTACGAGCTGACTCAAGAGTTCTTCCAAAATGGGAAGCCGGTTTGTGCTGAGAGCCAGAACAGCGTTGGCGTTTTTACTCGAGATGT
ENSORLG00000007567	0	klhl43	0.23	reference	wavelength	TGGCTTTCCAGCTTGCCATGAAGTGGCTGGACTTTAACGCCTCCCGCCAGGCTCACGCTTCTGACCTTCTGTCACATATTCGTTTTGAGACCATCCCAGC
ENSORLG00000009285	0	txlnb	-0.50	reference	wavelength	AGGTCCTCTACGACAAGATCAAGGAAGTTTGCCAGTCCAACGCCAGCCTTCCATCAAAGCTCGCTGAGCTTGTTGAGCCAAACAACGTTGCTAACGAAGC
ENSORLG00000009612	0	lepa	-1.26	reference	wavelength	AGCATCATACAGACTTCTATTGGCCCAAAGTTCTCGCCACCTTCTGATGAGCTGAATGGACTTTCCTCCATAATGGCCGTCTTAGATGAATGCACTAACC
ENSORLG00000016928	0	prl	0.53	reference	wavelength	CTGCTCAAAGCCTGGCAGTATCCCGTGGGGGTCCTGTCCAACTCTGCAAACTCGCTGCCTCACCAATCCAAGGGCAGCATTTCCAACAAGCTCCAGGAGC
ENSORLG00000019556	0	gh1	-0.04	reference	wavelength	TGATCAGGGCCAATCAGAATGTGGCTGAGATATATGAAGACACCCTGTCTCTTCCACTGGGGGAGTACATAGACTATTATCATGGACTTGGAGCCGATGG

ENSORLG00000020401	0	mfap4	-1.19	reference	wavelength	TGCAAACCCTAACGGCATTTATCGCTGGGGGAGAGATGATTACTTCAATTCAATTGGAGTGGAGTGGGATAACTGGAAGGGTCAGAACTATTCTCTGAAG
